# Impacts of Acute Hypoxia on Alzheimer's Disease-Like Pathologies in APP^swe^/PS1^dE9^ Mice and Their Wild Type Littermates

**DOI:** 10.3389/fnins.2018.00314

**Published:** 2018-05-09

**Authors:** Feng Zhang, Rujia Zhong, Hongqian Qi, Song Li, Cheng Cheng, Xinyao Liu, Yufei Liu, Weidong Le

**Affiliations:** ^1^Center for Clinical Research on Neurological Diseases, The First Affiliated Hospital, Dalian Medical University, Dalian, China; ^2^Liaoning Provincial Key Laboratory for Research on the Pathogenic Mechanisms of Neurological Diseases, The First Affiliated Hospital, Dalian Medical University, Dalian, China; ^3^Collaborative Innovation Center for Brain Science, The First Affiliated Hospital, Dalian Medical University, Dalian, China

**Keywords:** Aβ, acute hypoxia, Alzheimer's disease, autophagy, mitochondria, tau phosphorylation

## Abstract

Alzheimer's disease (AD) is the most common form of dementia and pathologically featured by β-amyloid (Aβ) plaque deposition and hyper-phosphorylated tau aggregation in the brain. Environmental factors are believed to contribute to the pathogenesis and progression of AD. In the present study, we investigated the impacts of acute hypoxia on Aβ and tau pathologies, neuroinflammation, mitochondrial function, and autophagy in APP^swe^/PS1^dE9^ AD mouse model. Male APP^swe^/PS1^dE9^ transgenic (Tg) mice and their age-matched wild type (Wt) littermates were exposed to one single acute hypoxic episode (oxygen 7%) for 24 h. We found that acute hypoxia exposure increased the expressions of amyloid precursor protein (APP), anterior pharynx-defective 1 (APH1) and cyclin-dependent kinase 5 (CDK5), and promoted tau phosphorylation at T181 and T231 residues in both Tg and Wt mice. In addition, acute hypoxia also induced autophagy through the mammalian target of rapamycin (mTOR) signaling, elicited abnormal mitochondrial function and neuroinflammation in both Tg and Wt mice. In summary, all these findings suggest that acute hypoxia could induce the AD-like pathological damages in the brain of APP^swe^/PS1^dE9^ mice and Wt mice to some extent.

## Introduction

Alzheimer's disease (AD) is the most common form of dementia and pathologically featured by amyloid β (Aβ) deposition and hyper-phosphorylated tau aggregation in the brain. Although the mechanisms underlying AD pathological changes are still unclear, Aβ and tau abnormality (Hardy and Selkoe, 2002; Iqbal et al., 2016), autophagy dysregulation (Li et al., 2010), mitochondrial dysfunction (Swerdlow et al., 2010) and neuroinflammation (Heppner et al., 2015) are believed to contribute to the pathogenesis of AD.

Aβ and tau pathologies are the pathological hallmarks of AD. Aβ, as the main component of the amyloid plaques in the AD brain, is derived from amyloid precursor protein (APP) processed sequentially by β- and γ-secretase (Thinakaran and Koo, 2008). After being cleaved, various different Aβ species are produced. While Aβ40 is the most abundant form of Aβ (about 80–90%), Aβ42 (about 5–10%) as principal form deposited in AD brain is more hydrophobic and fibrillogenic and is more prone to the synaptic damage, neuritic injury and neuronal death (Murphy and LeVine, 2010). Tau is a microtubule associated protein which binds to microtubules and stabilizes their structure (Giacobini and Gold, 2013). Hyper-phosphorylation of tau is caused by an imbalanced regulation of kinases and phosphatases, including glycogen synthase kinase-3β (GSK-3β), cyclin-dependent-like kinase-5 (CDK5) and protein phosphatase 2A (PP2A) (Sontag et al., 2007; Iqbal et al., 2016). Hyper-phosphorylated tau is unable to interact with microtubules and results in disruption of axonal flow. Additionally, the hyper-phosphorylated tau promotes its self-assembly into paired helical filaments (PHFs), which aggregates into neurofibrillary tangles (NFTs) (Giacobini and Gold, 2013).

Besides Aβ and tau pathologies, dysregulated autophagy, abnormal mitochondrial function and neuroinflammation are also thought to be involved in AD pathogenesis. Autophagy is a complex lysosome-mediated degradation system that is essential to clear misfolded or aggregated proteins and dysfunctional or damaged organelles (Murrow and Debnath, 2013). Electron microscopy observation usually illustrates an accumulation of autophagosomes in different autophagic stages, implying a disturbed autophagy in AD brains (Nilsson and Saido, 2014). The mechanistic target of rapamycin (mTOR) signaling pathway is one of the pathways negatively regulating autophagy. Most autophagy-inducing conditions such as nutrient/growth factor deprivation and low cellular energy have inhibitory effects on mTOR activity (Kim and Guan, 2015). Mitochondrial abnormalities are also characteristic of AD and etiologically involved in the brain degenerative process (Hu et al., 2017). Mitochondria in AD brain has been reported to have reduced membrane potential, increased permeability, and produce excessive reactive oxygen species (ROS) which are toxic to neurons and cause neurodegeneration (Onyango et al., 2016). An increased mitochondrial fission and mitophagy are also observed in astrocytes and glial cells, which play important roles in the pathogenesis of AD by participating in neuroinflammation (Motori et al., 2013). Moreover, Aβ and NFTs can also activate immune response and lead to the release of inflammatory cytokines, chemokines, and neurotoxins which may contribute to the neuronal degeneration (Calsolaro and Edison, 2016).

Although the etiology of AD is still far from being clarified, it is generally considered that most cases of AD especially sporadic AD arise through the interactions between genetic and environmental factors. Additionally, increasing evidence has suggested that AD results from a complex perturbance of systematic changes induced by multiple pathological processes rather than one single overriding factor. Hypoxia, as one of the important environmental risk factors, is reported to contribute to the onset and progression of AD (Zhang et al., 2013). Previous studies indicated that hypoxia might increase Aβ production (Li et al., 2009), enhance tau phosphorylation (Gao et al., 2013) and induce neuroinflammation (Smith et al., 2013). Furthermore, hypoxia might increase ROS (Zhang and Le, 2010). The mitochondria-derived ROS can activate hypoxia inducible factor-1α (HIF-1α) through different mechanisms including post-translational modification (Bruick and McKnight, 2001), and p38 mitogen-activated protein kinase (Emerling et al., 2005).

APP^swe^/PS1^dE9^ transgenic (Tg) mouse model is most widely used AD mouse model. It contains a chimeric mouse/human APP (Mo/HuAPP695swe) and a mutant human presenilin 1 (PS1-dE9), which resemble an early-onset AD. According to the description of Jackson Laboratory (https://www.jax.org/strain/004462), the Mo/HuAPP695swe transgene allows the mice to secrete a human Aβ peptide and the transgenic mice develop Aβ deposits in the brains by 6–7 months of age. Because female Tg mice are influenced by physiological cycles and hormone levels and have variable pathological outcome, male Tg mice are mostly used in many experimental studies (van Duijn et al., 2013; Jiao et al., 2016). In the present study, we used male Tg mice and their wild type (Wt) littermates to investigate the impacts of acute hypoxia on AD pathologies, which may provide a better understanding of the pathological roles of acute hypoxia in AD.

## Materials and methods

### Animals and hypoxic treatment

Adult male APP^swe^/PS1^dE9^ Tg mice at the age of 6 months and their age-matched Wt littermates were included in the present study. Tg mice were purchased from the Jackson Laboratory (No. 004462, Bar Harbor, MA, USA). All animals were housed under the condition of controlled light (12/12 h light/dark cycle, lights on at 8 AM), constant room temperature (22 ± 1°C) and relative humidity (50 ± 10%). The mice were randomized into four groups, with 10 mice in each group: Tg mice with acute hypoxia (H-Tg), Tg mice with normoxia (N-Tg), Wt mice with acute hypoxia (H-Wt), and Wt mice with normoxia (N-Wt). The animals in hypoxia groups were exposed to a continued hypoxic condition (oxygen 7%) in a hypoxic chamber for 24 h as described in our previous study (Zhang et al., 2017). The mice of normoxia groups were kept in a similar chamber with normoxic condition. After hypoxic exposure, the mice were immediately sacrificed for pathological and biochemical analysis. Hippocampus and temporal cortex adjacent to the hippocampus were chosen in the present study. Animal care and procedures were carried out in accordance with the Laboratory Animal Care Guidelines approved by the Institutional Animal Care Committee at Dalian Medical University. The protocol was approved by the Institutional Animal Care Committee at Dalian Medical University.

### Western blotting analysis

Western blotting was performed according to our previous protocols (Liu et al., 2015, 2016; Qiu et al., 2016). Tissues of hippocampus and cortex (*n* = 5 in each group) were dissected rapidly on ice and sonicated in ice-cold lysis buffer (containing 50 mM Tris pH 7.4, 150 mM sodium chloride, 1% Triton X-100, 1% sodium deoxycholate, 0.1% sodium dodecyl sulfonate, and phosphatase inhibitors such as sodium orthovanadate, sodium fluoride, ethylene diamine tetraacetic acid, leupeptin, etc. P10013B, Beyotime Institute of Biotechnology, China). The lysate was centrifuged at 12,000 × g for 10 min at 4°C. Then the supernatant fraction was collected for Western blotting analysis. Nucleoproteins and cytoplasmic proteins were extracted with Nucleoprotein and Cytoplasmic Protein Extraction Kit (KGP150, KeyGEN BioTECH, China). Mitochondrial and cytosolic proteins were extracted with Tissue Mitochondia Isolation Kit (C3606, Beyotime Institute of Biotechnology, China). BCA Protein Assay Kit (T9300A, Takara, Shiga, Japan) was used to detect the protein concentration. The primary antibodies used in Western blotting analysis were as follow: Glyceraldehyde 3-phosphate Dehydrogenase (GAPDH) (14C10) Antibody (2118S, 1:1,000; Cell Signaling, Chicago, IL, USA); β-Tubulin (9F3) Antibody (2128S, 1:5,000; Cell Signaling, Chicago, IL, USA); Anti-APP Antibody (Y188) (ab32136, 1:10,000; Abcam, Cambridge, UK); Anti-β-site APP Cleaving Enzyme 1 (BACE1) Antibody (ab2077, 1:1,000; Abcam, Cambridge, UK); Anti-Presenilin1 (PS1) Antibody (EP2000Y) (ab76083, 1:5,000; Abcam, Cambridge, UK); Anti-Presenilin Enhancer 2 (PEN2) Antibody (ab18189, 1:500; Abcam, Cambridge, UK); Anterior Pharynx-Defective 1A (APH1A) Antibody (11643-1-AP, 1:500; Proteintech, Rosemont, IL, USA); Anti-Nicastrin (NCSTN) Antibody (ab122969, 1:1,000; Abcam, Cambridge, UK); Tau Monoclonal Antibody (T46) (13-6400, 1:1,000; Thermo Fisher Scientific, Waltham, MA, USA); Anti-Tau (phospho Thr181) (D9F4G) Antibody (12885S, 1:1,000; Cell Signaling, Chicago, IL, USA); Anti-Tau (phospho T231) Antibody (EPR2488) (ab151559, 1:1,000; Abcam, Cambridge, UK); Anti-Tau (phospho S396) Antibody (EPR2731) (ab109390, 1:10,000; Abcam, Cambridge, UK); CDK5 Antibody (2506S, 1:500; Cell Signaling, Chicago, IL, USA); p35/25 (C64B10) Antibody (2680S, 1:1,000; Cell Signaling, Chicago, IL, USA); Anti-GSK3β Antibody (Y174) (ab32391, 1:5,000; Abcam, Cambridge, UK); Anti-GSK3β (phospho Y216) Antibody (ab75745, 1:1,000; Abcam, Cambridge, UK); Microtubule-associated Protein 1A/1B-light Chain 3B (LC3B) Antibody (2775S, 1:1,000; Cell Signaling, Chicago, IL, USA); Sequestosome 1 (SQSTM1)/p62 Antibody (5114S, 1:1,000; Cell Signaling, Chicago, IL, USA); Anti-mTOR Antibody (Y391) (ab32028, 1:2,000; Abcam, Cambridge, UK); Anti-mTOR (phospho S2448) Antibody [EPR426(2)] (ab109268, 1:1000; Abcam, Cambridge, UK); P70 Ribosomal Protein S6 Kinase (P70S6K) (49D7) Antibody (2708S, 1:1,000; Cell Signaling, Chicago, IL, USA); Phospho-P70S6K (Ser371) Antibody (9208S, 1:1,000; Cell Signaling, Chicago, IL, USA); Cytochrome C (Cyt C) Antibody (4272S, 1:1,000; Cell Signaling, Chicago, IL, USA); Anti-Cytochrome C Oxidase Subunit IV (COX IV) Antibody (20E8C12) (ab14744, 1:1,000; Abcam, Cambridge, UK); HIF-1α Antibody (NB100-105, 1:500; Novus Biologicals, USA). The secondary antibodies were Anti-Rabbit/Mouse IgG, HRP-linked antibody (7076/7074, 1:2,000; Cell Signaling, Chicago, IL, USA). The target protein bands were quantified by using FluorChem Q system (ProteinSimple, California, USA). The uncropped Western blotting pictures were provided in the Data Sheet in Supplementary Material.

### Immunostaining

For histological analysis, mice were anesthetized and perfused transcardially with cold phosphate buffer solution (PBS) and 4% paraformaldehyde (PFA). The whole brains were post-fixed with 4% PFA overnight and then dehydrated in 15 and 30% sucrose solutions. The brain tissues were then coated with Tissue-Tek optimal cutting temperature compound (O.C.T., Tissue-Tek, 4583, SAKURA, Torrance, USA). All brain tissues were cut coronally into 10 μm coronal sections with Leica cryostat (CM-1950S, Leica, Germany). The slices were used for phosphorylated tau (p-tau) 231 (ab151559, 1:200 Abcam, Cambridge, UK) and LC3 (2775S, 1:200, Cell Signaling, Chicago, IL, USA) staining. The secondary antibodies were Anti-Rabbit IgG (H+L), F(ab')_2_ Fragment (Alexa Fluor® 594 Conjugate) (8889S, 1:2,000; Cell Signaling, USA). Pictures were visualized and photographed by a fluorescent microscope equipped with a DP80 CCD digital camera (Olympus, Tokyo, Japan). Three microscopic fields, 0.1 mm^2^ per slice were captured with the same reference position of hippocampus. The integrated density of positive staining was measured and recorded by Image J software on 10 slices per animal (n = 3 in each group).

### Enzyme-linked immunosorbent assay

Tissues of hippocampus and cortex were dissected, weighed and sonicated in ice-cold lysis buffer. The lysate was centrifuged at 12,000 × g for 10 min at 4 °C. The supernatant was collected for measuring human Aβ40, human Aβ42, mouse Aβ40, and mouse Aβ42 (pg/mg protein) according to the manufacturer's instruction by using ELISA kits (Human Aβ40 ELISA Kit, KHB3482; Ultrasensitive human Aβ42 ELISA Kit, KHB3544; mouse Aβ40 ELISA Kit, KMB3481; mouse Aβ42 ELISA Kit, KMB3441; Thermo Fisher Scientific, Waltham, MA, USA). The ratio of Aβ42–Aβ40 was further calculated.

### Gene expression assessment

Protocols for total RNA extraction, cDNA synthesis and quantitative real-time polymerase chain reaction (PCR) were described previously (Tang et al., 2014). Mice were sacrificed immediately after the 24-h hypoxia episode. Tissues of hippocampus and cortex (*n* = 4 in each group) were dissected and extracted for total RNA with RNAiso Plus (Total RNA extraction reagent) (Takara, Shiga, Japan). According to Revertra Ace qPCR RT kit (Takara, Shiga, Japan) instructions, total RNA was synthesized to cDNA. Real-time PCR was performed with TransStart Top Green qPCR SuperMix (TransGen Biotech, Beijing, China) and monitored by the Real-time PCR System (Applied Biosystems 7500 Real-Time PCR Systems). The primers sequences were summarized in Table 1. The relative expression levels of each primers sequences mRNA were analyzed by the 2^−ΔΔCt^ algorithm normalizing to GAPDH and relative to the control groups.

**Table 1:** Primers sequences for real-time PCR.

	**Forward (5′-3′)**	**Reverse (5′-3′)**	**Gene access number**
GAPDH	TGTGTCCGTCGTGGATCTGA	TTGCTGTTGAAGTCGCAGGAG	NM_001289726.1
CD86	ACGATGGACCCCAGATGCACCA	GCGTCTCCACGGAAACAGCA	NM_019388.3
CD206	TCAGCTATTGGACGCGAGGCA	TCCGGGTTGCAAGTTGCCGT	NM_008625.2
IL-4	ATGGGTCTCAACCCCCAGCTAGT	TGCATGGCGTCCCTTCTCCTGT	NM_021283.2
IL-6	AACGATAGTCAATTCCAGAAACCG	GACAGGTCTGTTGGGAGTGG	NM_031168.2
IL-10	GGCAGAGAAGCATGGCCCAGAA	AATCGATGACAGCGCCTCAGCC	NM_010548.2
TNF-α	CCAGTGTGGGAAGCTGTCTT	AAGCAAAAGAGGAGGCAACA	NM_013693.3
CCL2	GCATCCACGTGTTGGCTCA	CTCCAGCCTACTCATTGGGATCA	NM_011333.3
CCL3	GCTCAACATCATGAAGGTCTCC	TGCCGGTTTCTCTTAGTCAGG	NM_011337.2

### Statistical analysis

All data were presented as mean ± standard error of the mean values (SEM). Statistical significance was determined using Student's *t*-test, two-way analysis of variance (ANOVA) with Tukey's multiple comparisons test by GraphPad Prism 7 (GraphPad Software Inc., California, USA). The results were considered to be statistically significant when *p*-value was less than 0.05. The *n* values in each figure legend represent the number of animals referred to the statistical analysis. Summarized statistical analysis data were shown in Supplementary Tables 1–4.

## Results

### Impact of acute hypoxia on Aβ production

**Figure 1:**
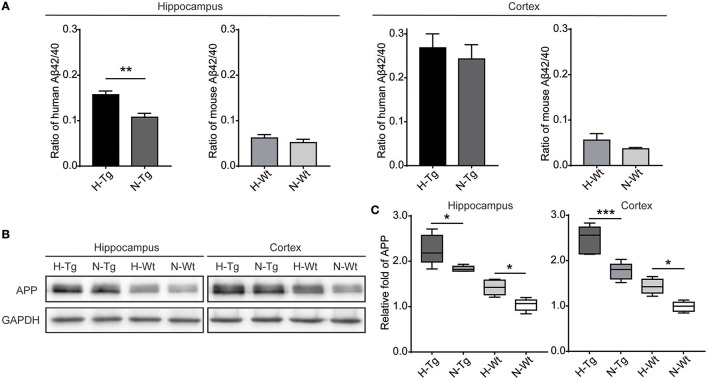
Amyloid β (Aβ)42/40 ratio and amyloid precursor protein (APP) protein level after acute hypoxia treatment. Human or mouse Aβ40 and Aβ42 levels were detected by human or mouse Enzyme-linked immunosorbent assay (ELISA) kit, Aβ42/40 ratio was increased significantly in hippocampus in H-Tg mice, *n* = 4 in each group **(A)**. Protein level of APP was increased significantly in both hippocampus and cortex in hypoxic mice, *n* = 5 in each group **(B,C)**. Data in **(A)** were shown as mean ± SEM. *p < 0.05, **p < 0.01, ***p < 0.001, Data in **(A)** were analyzed by Student's *t*-test. Data in **(B,C)** were analyzed by two-way ANOVA with Tukey's multiple comparisons test.

**Figure 2:**
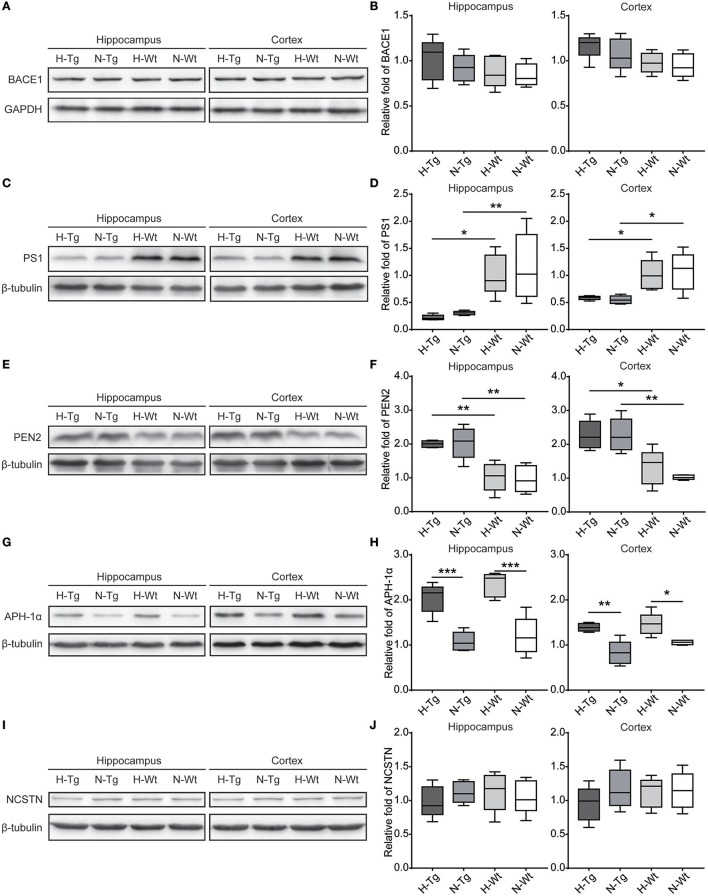
Protein levels of β-secretase and components of γ-secretase in the hippocampus and cortex after acute hypoxia. Protein level of component of γ-secretase, Anterior pharynx-defective 1 (APH-1α), was increased significantly in the hippocampus and cortex of hypoxic mice **(G,H)**. β-secretase (BACE1) **(A,B)**, presenilin1 (PS1) **(C,D)**, presenilin enhancer 2 (PEN2) **(E,F)** and nicastrin (NCSTN) **(I,J)** showed unchanged levels between hypoxic and normoxic mice. *n* = 5 mice in each group. *p < 0.05, **p < 0.01, ***p < 0.001, by two-way ANOVA with Tukey's multiple comparisons test.

First, we measured the nuclear protein level of hypoxia inducible factor-1α (HIF-1α) *in vivo*, showing that nuclear HIF-1α was increased in the hippocampus and cortex in both Tg and Wt mice after acute hypoxic treatment (Supplementary Figure 1). To investigate the possible impact of acute hypoxia on Aβ pathology, we firstly detected the Aβ40 and Aβ42 levels in hippocampus and cortex. The results showed that Aβ42/40 ratio was increased significantly in hippocampus in H-Tg mice when compared to N-Tg mice (Figure 1A, left). In order to interpret the possible mechanisms for the altered Aβ42/40 ratio, we next measured the protein expression levels of APP, β-secretase and components of γ-secretase including PS, NCSTN, APH1, and PEN2. We found that APP level was increased significantly in both hippocampus and cortex in hypoxia-treated mice (Figures 1B,C). Furthermore, APH-1α level was also increased significantly in hypoxia-treated mice compared to normoxic controls, respectively (Figure 2). These results indicate that acute hypoxia is able to induce Aβ metabolic abnormality via enhancing APP expression and promoting the γ-secretase cleavage of APP.

### Impact of acute hypoxia on tau phosphorylation

**Figure 3:**
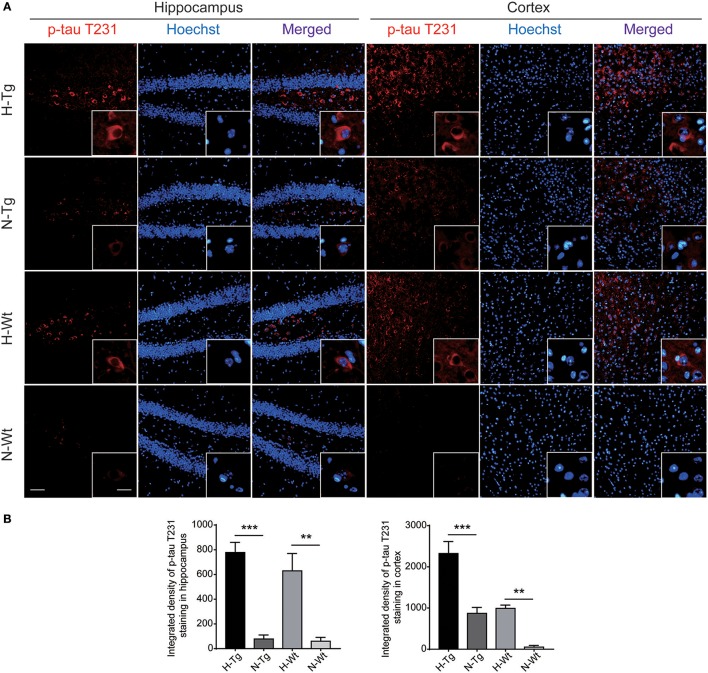
Immunoflourescent staining of phosphorylated tau (p-tau) T231 in the mouse hippocampus and cortex after acute hypoxia. The p-tau T231 staining was increased significantly in the hippocampus and cortex of hypoxic mice when compared to normoxic mice **(A)**. Integrated density of p-tau T231 staining was analyzed **(B)**. Left scale bar: 50 μm, right scale bar: 20 μm, *n* = 3 mice in each group. Data were the mean ± SEM values. **p < 0.01, ***p < 0.001, by two-way ANOVA with Tukey's multiple comparisons test.

**Figure 4:**
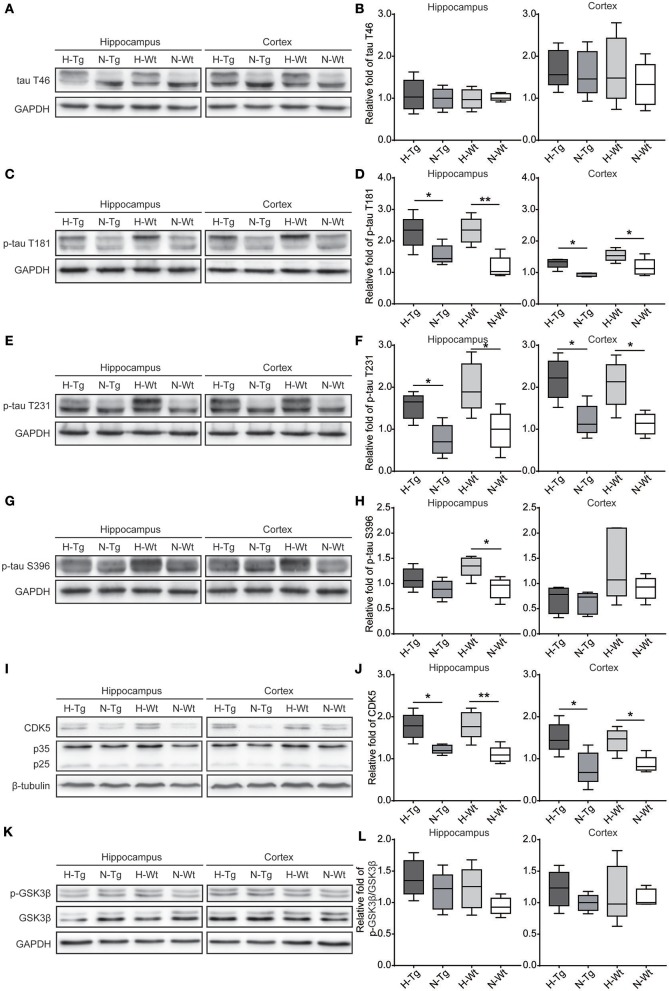
Protein levels of total tau (t-tau), p-tau and its regulating kinases in the hippocampus and cortex after acute hypoxia. Protein levels of p-tau at T181 **(C,D)**, T231 **(E,F)**, S396 **(G,H)** and cyclin-dependent-like kinase-5 (CDK5) and its activator p35 and p25 **(I,J)** were increased in the hippocampus and cortex of hypoxic mice. Changes in total-tau **(A,B)**, glycogen synthase kinase-3β (GSK-3β)/phosphorylated GSK-3β (p-GSK-3β) ratio **(K,L)** were moderate. *n* = 5 mice in each group. *p < 0.05, **p < 0.01, by two-way ANOVA with Tukey's multiple comparisons test.

As for tau pathology, we firstly performed immunofluorescence imaging of p-tau T231 in Tg and Wt mouse hippocampus and cortex. As shown in Figure 3, p-tau T231 staining was significantly increased in both H-Tg and H-Wt mice. Quantitative analysis further confirmed that the integrated density of p-tau T231 staining was increased significantly in hypoxic mice. Then we detected total tau (t-tau) level and p-tau levels at T181, T231, S396 residues by Western blotting. Levels of p-tau T181, T231 were increased significantly in both hippocampus and cortex in H-Tg and H-Wt mice when compared to N-Tg and N-Wt mice, respectively. Changes of p-tau S396 level seemed moderate (Figure 4). The ratios of p-tau T181/t-tau, p-tau T231/t-tau and p-tau S396/t-tau showed similar profiles (Supplementary Figure 2). Moreover, we also investigated the levels of protein kinase that regulated tau phosphorylation. We found that CDK5 level was increased significantly in hippocampus and cortex in hypoxia groups (Figures 4I,J). However, the levels of phosphorylated GSK3β (p-GSK3β) and GSK3β remained unchanged (Figures 4K,L).

### Impact of acute hypoxia on autophagy

**Figure 5:**
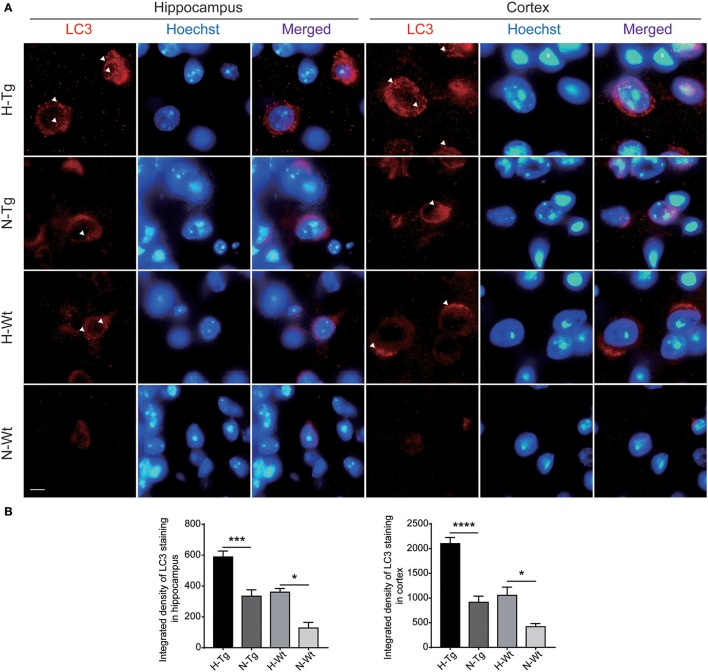
Immunoflourescent staining of microtubule-associated protein 1A/1B-light chain 3 (LC3) in the mouse hippocampus and cortex after acute hypoxia. The LC3 staining was increased significantly in the hippocampus and cortex of hypoxic mice when compared to normoxic mice **(A)**. Integrated density of LC3 staining was analyzed **(B)**. Arrow heads showed LC3 puncta. Scale bar: 10 μm, *n* = 3 mice in each group. Data were the mean ± SEM values. *p < 0.05, ***p < 0.001, ****p < 0.0001, by two-way ANOVA with Tukey's multiple comparisons test.

**Figure 6:**
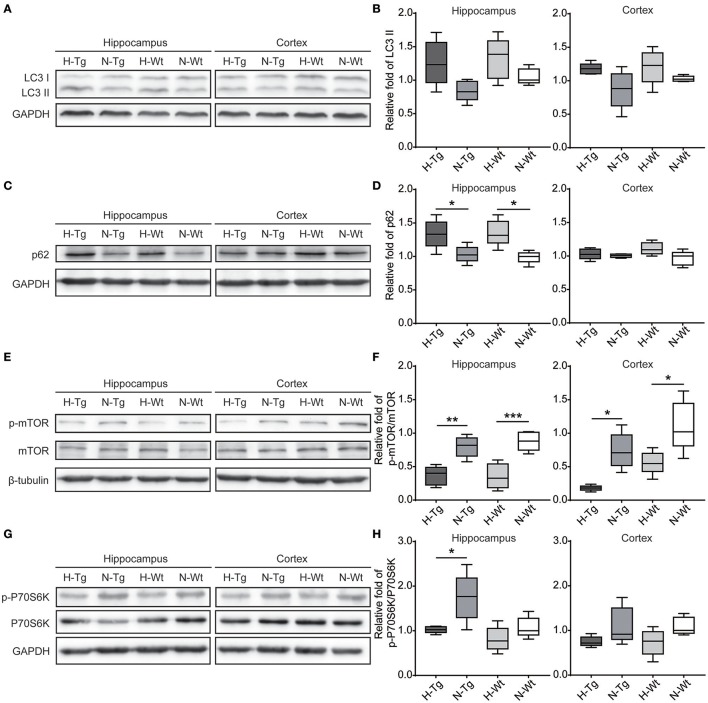
Protein levels of LC3, p62 and proteins in mechanistic target of rapamycin (mTOR) signaling in the hippocampus and cortex after acute hypoxia. LC3 protein level showed an increase in hypoxic mice **(A,B)**. p62 was increased significantly in the hippocampus of hypoxic mice **(C,D)**. Ratio of phosphorylated mechanistic target of rapamycin (p-mTOR)/mTOR **(E,F)** and ratio of phosphorylated p70 ribosomal protein S6 kinase (p-P70S6K)/P70S6K **(G,H)** were decreased in hypoxic mice. *n* = 5 in each group. *p < 0.05, **p < 0.01, ***p < 0.001, by two-way ANOVA with Tukey's multiple comparisons test.

To determine autophagy level in the brains of those hypoxia-treated Tg and Wt mice, we firstly detected LC3 level by immunofluorescence. LC3 II is usually considered as a marker of autophagosome in the early induction of autophagy (Liu et al., 2015). As shown in Figure 5, LC3 staining was increased in both hippocampus and cortex in H-Tg and H-Wt mice, compared with N-Tg and N-Wt mice, respectively. Quantitative analysis further confirmed that the integrated density of LC3 staining was increased significantly in hypoxic mice. Protein levels of LC3, p62 were also examined by western blotting, showing an increase of LC3 II level in both hippocampus and cortex and an increase of p62 level in hippocampus (Figure 6A-D). These results indicate that autophagy is activated after acute hypoxia exposure. Further, we detected protein levels of mTOR, P70S6K and their phosphorylated forms to determine whether the hypoxia-induced autophagic activation was via mTOR pathway (Shinojima et al., 2007) (Figures 6E–H). P70S6K is a downstream protein kinase of mTOR, which can be phosphorylated and activated by mTOR (Ravikumar et al., 2010). Western blotting results showed that the ratio of phosphorylated mTOR (p-mTOR)/mTOR was decreased significantly in hypoxic mice compared to normoxic mice, indicating an inhibited mTOR signaling. Ratio of phosphorylated P70S6K (p-P70S6K)/P70S6K was decreased significantly in the hippocampus in H-Tg mice when compared to N-Tg mice. These results indicate that acute hypoxia could activate autophagy through the inhibition of mTOR signaling pathway.

### Impact of acute hypoxia on mitochondrial function

**Figure 7:**
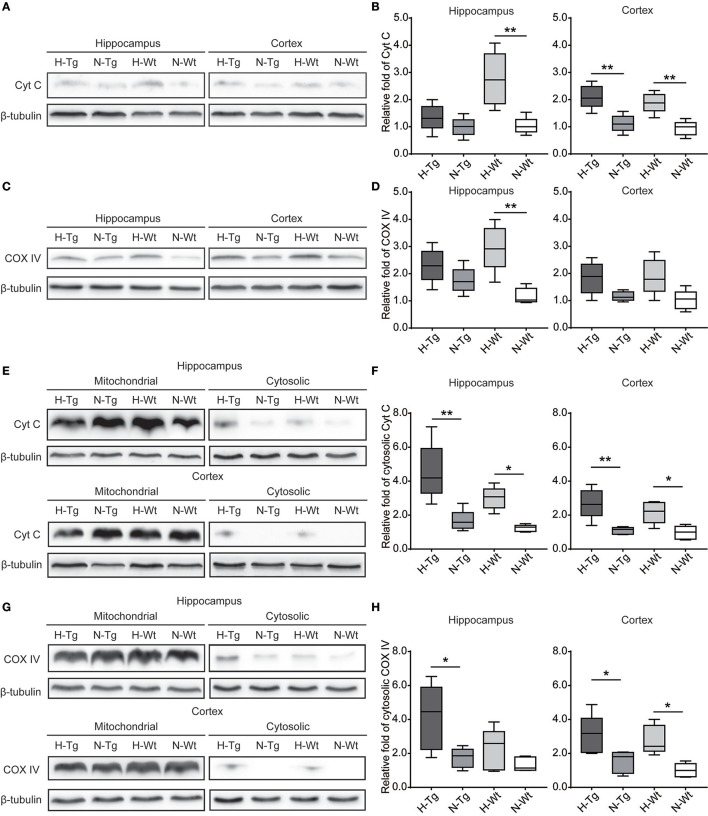
Protein levels of cytochrome c (Cyt C) and cytochrome c oxidase subunit IV (COX IV) in the hippocampus and cortex after acute hypoxia. Cyt C **(A,B)** and COX IV **(C,D)** total protein levels and Cyt C **(E,F)** and COX IV **(G,H)** protein levels in cytosolic fraction were increased in the hippocampus and cortex of hypoxic mice. *n* = 5 mice in each group. *p < 0.05, **p < 0.01, by two-way ANOVA with Tukey's multiple comparisons test.

To investigate the impact of acute hypoxia on mitochondrial function, we detected protein levels of Cyt C and COX IV. As shown in Figure 7, western blotting results showed that Cyt C protein level was significantly increased in the hippocampus of H-Wt mice compared to N-Wt mice and in the cortex of both H-Tg and H-Wt mice when compared to N-Tg and N-Wt mice, respectively. Consistently, COX IV was increased significantly in hippocampus of H-Wt mice compared to N-Wt mice. Further, we measured the protein levels of Cyt C and COX IV in mitochondrial and cytosolic fraction. Cytosolic Cyt C and COX IV were increased in both hippocampus and cortex of hypoxic mice.

### Impact of acute hypoxia on neuroinflammation

**Figure 8:**
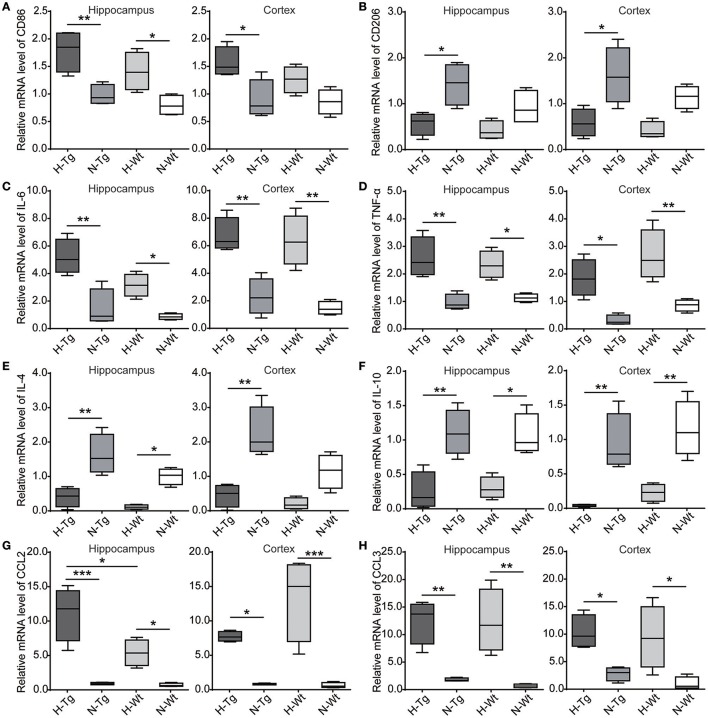
mRNA levels of cytokines and chemokines were detected by real-time PCR in the hippocampus and cortex after acute hypoxic treatment. M1 marker cluster of differentiation 86 (CD86) **(A)**, pro-inflammatory cytokines interleukin-6 (IL-6) **(C)** and tumor necrosis factor-α (TNF-α) **(D)**, chemokines C-C motif ligand 2 (CCL2) **(G)** and CCL3 **(H)** were increased in the hypoxic mice whereas M2 marker CD206 **(B)**, anti-inflammatory cytokines IL-4 **(E)** and IL-10 **(F)** were decreased in the hypoxic mice. *n* = 4 mice in each group. *p < 0.05, **p < 0.01, ***p < 0.001, by two-way ANOVA with Tukey's multiple comparisons test.

We determined microglia markers, inflammatory cytokines and chemokines in both hippocampus and cortex on the mRNA level (Figure 8). We found that the pro-inflammatory M1 marker cluster of differentiation 86 (CD86), along with pro-inflammatory cytokines and chemokines, including interleukin-6 (IL-6), tumor necrosis factor-α (TNF-α), chemokine C-C motif ligand 2 (CCL2) and CCL3, were increased in both hippocampus and cortex in hypoxic mice compared to normoxic mice. The immunosuppressive M2 marker CD206, anti-inflammatory cytokines including IL-4 and IL-10 were decreased in the hippocampus and cortex of hypoxic mice.

## Discussion

Hypoxia has been reported as one of the important environmental risk factors of AD. Many studies have confirmed that hypoxia facilitates AD pathological changes (Shiota et al., 2013; Liu et al., 2015; Yagishita et al., 2017; Zhang et al., 2017). In the present study, we demonstrated the impacts of acute hypoxia on AD-like pathologies including Aβ and tau pathologies, dysregulated autophagy, mitochondrial dysfunction and neuroinflammation.

First, our results showed that acute hypoxia could change the Aβ production through the altered expressions of APP and γ-secretase component APH1. These findings were consistent with the data from our previous study (Liu et al., 2016), in which we documented that chronic hypoxia downregulated DNA methyltransferase 3b and lowered methylated CpG sites in promoter regions of PS1, PEN2, and NCSTN genes. Other studies have reported that chronic hypoxia increased Aβ generation through the upregulated BACE1 and hypoxia inducible factor-1α (HIF-1α) expressions. Binding of HIF-1α to its promoter might result in over-expression of BACE1 (Sun et al., 2006; Zhang et al., 2007). In the present study, we only observed an increased level of γ-secretase component but not BACE1 protein after acute hypoxia exposure. This inconsistency implied that there might be different regulating mechanisms underlying the hypoxia-induced Aβ generation between acute and chronic hypoxia conditions.

Second, it is well known that CDK5 and GSK3β are two major kinases contributing to the abnormal phosphorylation of tau in AD brain (Kimura et al., 2014). We demonstrated that acute hypoxia increased p-tau at T181 and T231 residues, and increased CDK5 level without significant changes in p-GSK3β and GSK3β. We also observed an increase of tau phosphorylation after chronic hypoxia treatment in our previous study (Liu et al., 2016). A study in rat found that 1, 2, 4, and 8 weeks of chronic hypoxia exposure increased tau phosphorylation at S198/199/202, T205, S262, S396, and S404 in the hippocampus along with increased Y216-phosphorylated GSK-3β (activated form of GSK-3β) and Y307-phosphorylated PP2A (inactivated form of PP2A) (Zhang et al., 2014). Another study reported that chronic hypoxia increased tau phosphorylation accompanied with the increased levels of CDK5, its activator p25 and calpain, and the increased level of p25 cleaved by calpain resulted from Aβ generation (Gao et al., 2013).

Third, our data showed that acute hypoxia activated autophagy in the brain of hypoxia-treated mice. Our previous study reported that chronic hypoxia induced autophagy through mTOR signaling, which aggravated the neuropathology of AD (Liu et al., 2015). Our present findings imply that acute hypoxia-induced autophagy might be associated with inhibition of mTOR/P70S6K signaling. It has been reported that ROS, such as superoxide and H_2_O_2_, could activate 5′ adenosine monophosphate-activated protein kinase (AMPK) through S-glutathionylation of reactive cysteines located at the α- and β-subunits of AMPK (Filomeni et al., 2015) which further led to phosphorylation and activation of the tuberous sclerosis complex1 (TSC1)/TSC2 and inhibition mTOR activity (Liu et al., 2015). This might be one of the mechanisms that contribute to the hypoxia-induced mTOR inhibition. Furthermore, we found an increased protein level of p62 in the hippocampus but not in the cortex of the hypoxic mice. p62 functions as a selective autophagy receptor for degradation of ubiquitinated substrates (Shinojima et al., 2007). The increase of p62 might indicate a disturbed autophagic flux in hippocampus. It is reported that autophagic vacuoles (AVs) contain the highest γ-secretase activity (Yu et al., 2005). Hypoxia activates autophagy and disturbs autophagic flux, which can lead to an accumulation of AVs and result in an excessive production of Aβ. All these previous data are consistent with our present findings. However, the inconsistent alterations in the autophagy function between hippocampus and cortex need further exploration.

Cyt C is an essential component of the electron transport chain in the mitochondrion, which transfers electrons to COX. COX IV is one of the subunits of COX complex. Excessive Ca^2+^ uptake into mitochondria or inhibition of electron transport chain increases ROS production, induces mitochondrial permeability transition pore (PTP), results in the releasing of small proteins, such as Cyt C, and triggers the initiation of apoptosis, which may be a mechanism of neurodegeneration in AD (Moreira et al., 2010). In present study, we documented the increased levels of Cyt C and COX IV in the hypoxic mouse brain, indicating a mitochondrial dysfunction after acute hypoxia.

Microglia act as the main form of active immune defense in central nervous system (CNS). It is generally considered that microglia can be activated as two different phenotypes: pro-inflammatory M1 and immunosuppressive M2. M1 microglia are associated with the releasing of pro-inflammatory cytokines and chemokines, including IL-1β, IL-6, TNF-α, CCL2, CCL3. M2 microglia are closely associated with the production of anti-inflammatory cytokines, such as IL-4, IL-10, and TGF-β (Tang and Le, 2016). As we previously reported, acute hypoxia elevated M1 activation and attenuated M2 activation along with increased pro-inflammatory cytokines and chemokines and decreased anti-inflammatory cytokines in hippocampus (Zhang et al., 2017). In the present study, we evaluated mRNA levels of M1/M2 markers, cytokines and chemokines in the hippocampus and cortex. The results were consistent with our previous study, indicating that acute hypoxia caused an imbalanced M1/M2 activation in the hippocampus and cortex.

In summary, we investigated the impacts of acute hypoxia on AD pathologies. We found that acute hypoxia collectively up-regulates Aβ production and tau phosphorylation, induces autophagy, elicited mitochondrial dysfunction and neuroinflammation. Except for human Aβ secretion, acute hypoxia has similar pathological effects on both Tg and Wt mice to some extent. Changes in Wt mice might represent hypoxic effects in sporadic AD. However, whether there are different mechanisms underlying the hypoxia-induced AD pathology between Tg and Wt mice require further investigation. These pathological changes may cause long-lasting consequence related to AD. For example, Aβ might induce tau phosphorylation (Gao et al., 2013) and tau is required for Aβ toxicity (Bloom, 2014). Autophagic dysfunction may lead to intraneuronal Aβ accumulation (Pickford et al., 2008), and Aβ and PS1 may regulate autophagy (Neely et al., 2011; Son et al., 2012). Increased mitochondrial fission and mitophagy may participate neuroinflammation (Hu et al., 2017) and excessive pro-inflammatory cytokines and chemokines may facilitate Aβ oligomer formation (Kiyota et al., 2009). All these pathological changes in combination may further contribute to the subsequent damage of brain. A single episode of acute hypoxia may cause neuronal dysfunction or even neuronal cell death in the brain. Many studies have demonstrated hypoxia as an important risk factor for AD (Savva et al., 2010; Gao et al., 2013). In our present study, a single episode of hypoxic condition was adopted. Clinical patients who have stroke or acute cerebral ischemia experience acute hypoxic condition, whereas patients with respiratory dysfunctions, such as obstructive sleep apnea syndrome (OSAS) and chronic obstructive pulmonary disease (COPD), usually undergo repeated hypoxic conditions. Our previous studies of chronic hypoxia have indicated that chronic hypoxia chronic hypoxia aggravated Aβ production though epigenetic modifications of γ-secretase (Liu et al., 2016) and altered autophagy (Liu et al., 2015), indicating the pathological roles of chronic hypoxia in AD progression. Further studies are needed to determine the underlying mechanisms of acute and chronic hypoxia on AD pathologies in both Tg and Wt mice. Overall, a better understanding of hypoxia in the pathogenesis of AD may help define the complex of the disease and provide new therapeutics and prevention targets for AD.

## Author contributions

WL designed the project of this manuscript. FZ and RZ carried out all the experiments. FZ, RZ, SL and HQ contributed to statistical analyses and results interpretation. FZ, RZ, SL, XL and YL contributed to drafting of the manuscript. HQ, RZ, SL, FZ, XL, YL, CC and WL revised the manuscript. WL contributed to research concept, research administration and support. All authors edited and approved the final version of the manuscript.

### Conflict of interest statement

The authors declare that the research was conducted in the absence of any commercial or financial relationships that could be construed as a potential conflict of interest.

## Supplementary material

Supplementary Figure 1 Nuclear and cytoplasmic protein levels of hypoxia inducible factor-1α (HIF-1α) in the hippocampus and cortex after acute hypoxia. Nuclear level of HIF-1α were increased in hypoxic mice in both hippocampus and cortex (A,B). n = 5 in each group. ^*^p < 0.05, ^**^p < 0.01, by two-way ANOVA with Tukey's multiple comparisons test.

Supplementary Figure 2 The ratios of p-tau T181/t-tau, p-tau T231/t-tau, and p-tau S396/t-tau in hippocampus and cortex after acute hypoxia. The ratio of p-tau T181/t-tau was increased in hippocampus (A). The ratio of p-tau T231/t-tau was increased in both hippocampus and cortex (B), The ratio of p-tau S396/t-tau was increased in hippocampus in H-Wt mice (C). n = 5 in each group. ^*^p < 0.05, ^**^p < 0.01, ^***^p < 0.001, ^****^p < 0.0001 by two-way ANOVA with Tukey's multiple comparisons test.

Supplementary Table 1 Summarized statistical analysis of ELISA.

Supplementary Table 2 Summarized statistical analysis of Western blotting.

Supplementary Table 3 Summarized statistical analysis of immunostaining.

Supplementary Table 4 Summarized statistical analysis of Gene expression assessment.
