# Supplementary figures and images for: Impacts of Acute Hypoxia on Alzheimer's Disease-Like Pathologies in APPswe/PS1dE9 Mice and Their Wild Type Littermates
Source: Front Neurosci. 2018 May 9;12:314. doi: 10.3389/fnins.2018.00314 (PMC5954115; doi:10.3389/fnins.2018.00314)

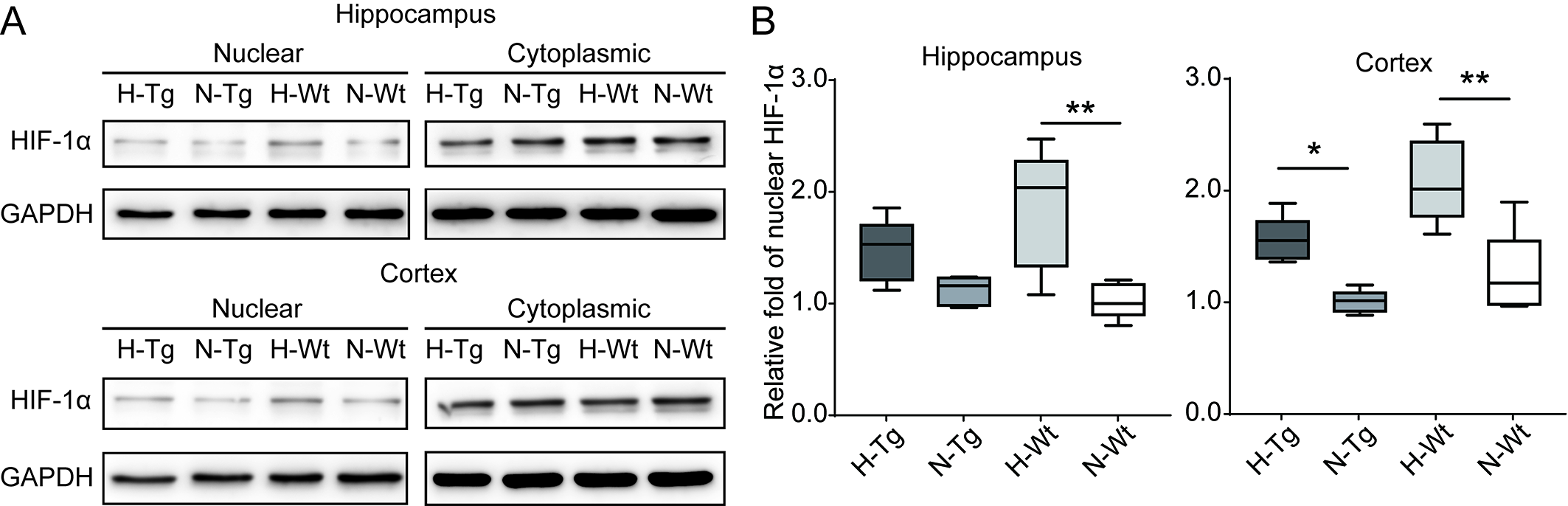

Supplement: Supplementary Figure 1 — Nuclear and cytoplasmic protein levels of hypoxia inducible factor-1α (HIF-1α) in the hippocampus and cortex after acute hypoxia. Nuclear level of HIF-1α were increased in hypoxic mice in both hippocampus and cortex (A,B). n = 5 in each group. *p < 0.05, **p < 0.01, by two-way ANOVA with Tukey's multiple comparisons test. [file Image_1.TIF]

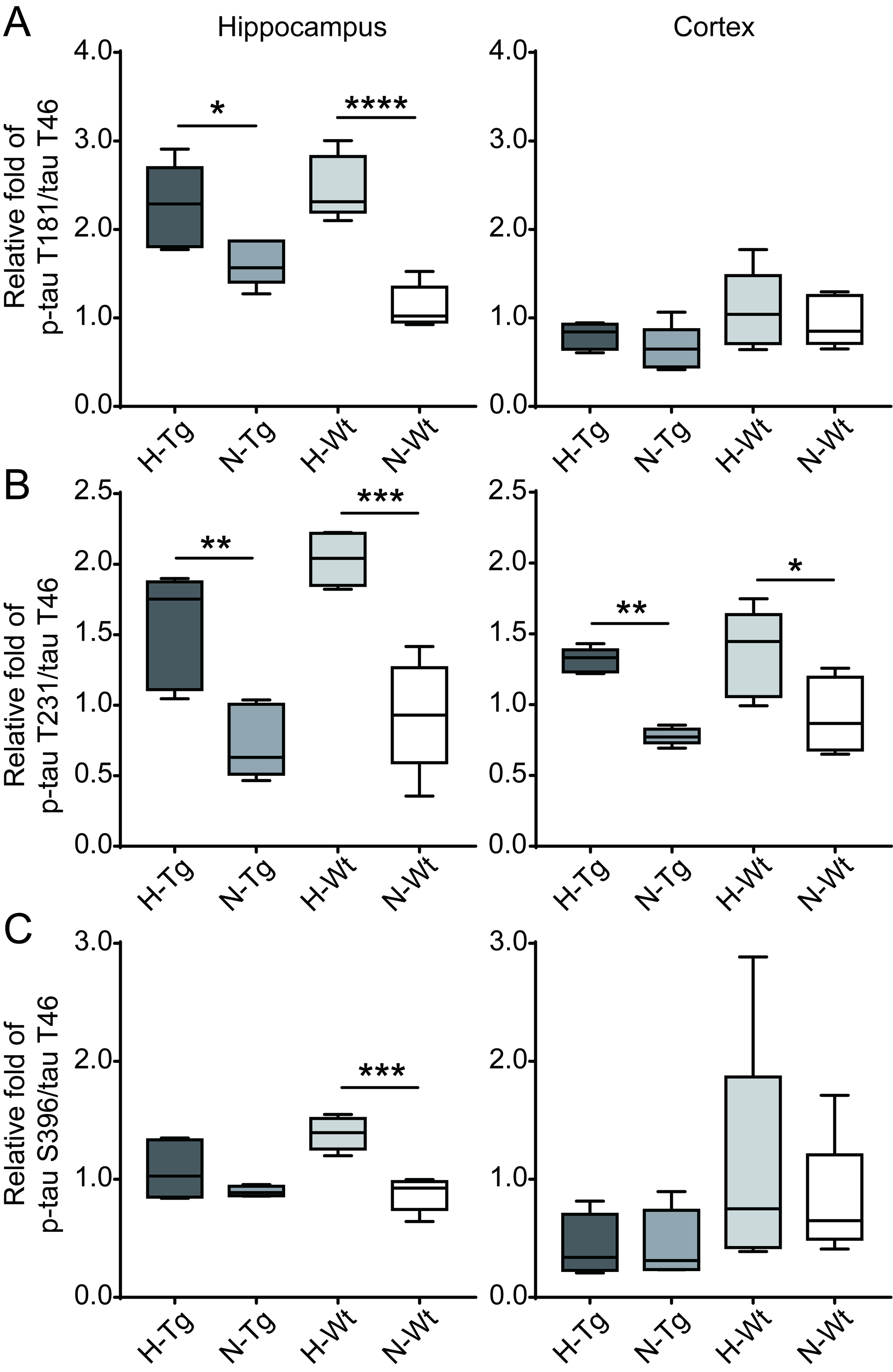

Supplement: Supplementary Figure 2 — The ratios of p-tau T181/t-tau, p-tau T231/t-tau, and p-tau S396/t-tau in hippocampus and cortex after acute hypoxia. The ratio of p-tau T181/t-tau was increased in hippocampus (A). The ratio of p-tau T231/t-tau was increased in both hippocampus and cortex (B), The ratio of p-tau S396/t-tau was increased in hippocampus in H-Wt mice (C). n = 5 in each group. *p < 0.05, **p < 0.01, ***p < 0.001, ****p < 0.0001 by two-way ANOVA with Tukey's multiple comparisons test. [file Image_2.TIF]
