# Supplementary material for: Impacts of Acute Hypoxia on Alzheimer's Disease-Like Pathologies in APPswe/PS1dE9 Mice and Their Wild Type Littermates
Source: Front Neurosci. 2018 May 9;12:314. doi: 10.3389/fnins.2018.00314 (PMC5954115; doi:10.3389/fnins.2018.00314)
Supplement: Supplementary Table 1 — Summarized statistical analysis of ELISA. [file Table_1.pdf]

SUPPLEMENTARY INFORMATION

Supplementary Table 1. Summarized statistical analysis of ELISA

	Hypoxia		Normoxia		t	df	P
	Mean	SEM	Mean	SEM			
Human Aβ42/40 in Tg mouse hippocampus	0.157	0.008242	0.1075	0.008636	4.146	6	0.0060
Mouse Aβ42/40 in Wt mouse hippocampus	0.06184	0.007615	0.05181	0.007228	0.9552	6	0.9552
Human Aβ42/40 in Tg mouse cortex	0.2683	0.0318	0.2429	0.0326	0.558	6	0.5970
Mouse Aβ42/40 in Wt mouse cortex	0.05586	0.01441	0.03675	0.002879	1.3	6	0.2414

Data were shown as the ratio of Aβ42 (pg/mg protein) to Aβ40 (pg/mg protein).

Supplementary Table 2. Summarized statistical analysis of Western blotting

	H-Tg		N-Tg		H-Wt		N-Wt		ANOVA SS				ANOVA F (DFn, DFd)			ANOVA P value		
	Mean	SEM	Mean	SEM	Mean	SEM	Mean	SEM	Interaction	Tg vs. Wt	H vs. N	Residual	Interaction	Tg vs. Wt	H vs. N	Interaction	Tg vs. Wt	H vs. N
APP hip	2.26	0.1476	1.834	0.02774	1.425	0.07394	1.042	0.05879	0.002281	3.307	0.8193	0.6294	F (1, 16) = 0.05799	F (1, 16) = 84.07	F (1, 16) = 20.83	P=0.8128	P<0.0001	P=0.0003
APP cor	2.475	0.137	1.775	0.08378	1.442	0.07376	0.9931	0.04853	0.07923	4.116	1.65	0.6714	F (1, 16) = 1.888	F (1, 16) = 98.08	F (1, 16) = 39.33	P=0.1884	P<0.0001	P<0.0001
BACE1 hip	1.014	0.1006	0.9188	0.03512	0.8771	0.06853	0.8401	0.05576	0.004286	0.05827	0.02195	0.4857	F (1, 16) = 0.1412	F (1, 16) = 1.92	F (1, 16) = 0.7231	P=0.7120	P=0.1849	P=0.4077
BACE1 cor	1.168	0.06278	1.072	0.08079	0.9794	0.05014	0.9794	0.0593	0.005143	0.1225	0.02061	0.3299	F (1, 16) = 0.2494	F (1, 16) = 5.943	F (1, 16) = 0.9992	P=0.6243	P=0.0268	P=0.3324
PS1 hip	0.2196	0.02223	0.3042	0.01618	1.015	0.1694	1.152	1.152	0.003469	3.374	0.06155	2.124	F (1, 16) = 0.02614	F (1, 16) = 25.42	F (1, 16) = 0.4638	P=0.8736	P=0.0001	P=0.5056
PS1 cor	0.5864	0.0162	0.5498	0.03277	1.011	0.1255	1.077	0.1582	0.005488	1.046	0.005488	0.826	F (1, 16) = 0.1063	F (1, 16) = 20.27	F (1, 16) = 0.1063	P=0.7486	P=0.0004	P=0.7486
PEN2 hip	2.01	0.04297	2.047	0.2098	1.036	0.187	0.9759	0.1747	0.01172	5.229	0.0006623	2.227	F (1, 16) = 0.08416	F (1, 16) = 37.56	F (1, 16) = 0.004757	P=0.7755	P<0.0001	P=0.9459
PEN2 cor	2.283	0.189	2.283	0.2205	1.332	0.2328	1.032	0.02985	0.1117	6.061	0.1125	2.789	F (1, 16) = 0.6405	F (1, 16) = 34.77	F (1, 16) = 0.6454	P=0.4352	P<0.0001	P=0.4335
APH-1α hip	2.045	0.1459	1.077	0.09382	2.347	0.1187	1.202	0.1867	0.03906	0.2275	5.585	1.581	F (1, 16) = 0.3953	F (1, 16) = 2.302	F (1, 16) = 56.53	P=0.5384	P=0.1487	P<0.0001
APH-1α cor	1.392	0.0388	0.8284	0.1171	1.463	0.1111	1.057	0.0221	0.03136	0.112	1.175	0.561	F (1, 16) = 0.8944	F (1, 16) = 3.195	F (1, 16) = 33.52	P=0.3583	P=0.0928	P<0.0001
NCSTN hip	0.9824	0.1037	1.122	0.07033	1.13	0.1282	1.059	0.1108	0.05538	0.009083	0.005785	0.8888	F (1, 16) = 0.9969	F (1, 16) = 0.1635	F (1, 16) = 0.1041	P=0.3329	P=0.6913	P=0.7511
NCSTN cor	0.9512	0.1147	1.174	0.1298	1.123	0.09815	1.147	0.1212	0.04929	0.02621	0.07581	1.087	F (1, 16) = 0.7256	F (1, 16) = 0.3859	F (1, 16) = 1.116	P=0.4069	P=0.5432	P=0.3065
tau T46 hip	1.076	0.1686	0.9913	0.1092	0.9794	0.1042	1.026	0.03838	0.02137	0.004733	0.001774	1.054	F (1, 16) = 0.3245	F (1, 16) = 0.07188	F (1, 16) = 0.02693	P=0.5769	P=0.7920	P=0.8717
tau T46 cor	1.69	0.2022	1.583	0.2416	1.663	0.3536	1.322	0.2319	0.06845	0.1039	0.251	5.561	F (1, 16) = 0.1969	F (1, 16) = 0.299	F (1, 16) = 0.7222	P=0.6632	P=0.5920	P=0.4080
p-tau T181 hip	2.282	0.2289	1.554	0.1378	2.326	0.1822	1.158	0.1506	0.2413	0.1543	4.496	2.545	F (1, 16) = 1.517	F (1, 16) = 0.9701	F (1, 16) = 28.26	P=0.2359	P=0.3393	P<0.0001
p-tau T181 cor	1.301	0.06943	0.9366	0.02484	1.542	0.08114	1.166	0.1188	0.0001878	0.2778	0.6846	0.5227	F (1, 16) = 0.005747	F (1, 16) = 8.502	F (1, 16) = 20.96	P=0.9405	P=0.0101	P=0.0003
p-tau T231 hip	1.553	0.1364	0.7464	0.163	2.003	0.264	0.9725	0.2074	0.06246	0.571	4.216	3.158	F (1, 16) = 0.3165	F (1, 16) = 2.893	F (1, 16) = 21.36	P=0.5815	P=0.1083	P=0.0003
p-tau T231 cor	2.198	0.2161	1.213	0.1668	2.081	0.2456	1.129	0.1134	0.00137	0.05028	4.687	2.954	F (1, 16) = 0.007423	F (1, 16) = 0.2723	F (1, 16) = 25.38	P=0.9324	P=0.6089	P=0.0001
p-tau S396 hip	1.097	0.09342	0.8825	0.08109	1.336	0.09228	0.9067	0.0922	0.05779	0.08681	0.5186	0.6464	F (1, 16) = 1.43	F (1, 16) = 2.149	F (1, 16) = 12.84	P=0.2491	P=0.1621	P=0.0025
p-tau S396 cor	0.681	0.1179	0.6228	0.0966	1.355	0.3139	0.9081	0.1011	0.1893	1.152	0.3194	2.64	F (1, 16) = 1.147	F (1, 16) = 6.979	F (1, 16) = 1.936	P=0.3000	P=0.0178	P=0.1832
CDK5 hip	1.773	0.1379	1.22	0.04652	1.801	0.1468	1.096	0.08627	0.02881	0.01118	1.977	1.003	F (1, 16) = 0.4595	F (1, 16) = 0.1784	F (1, 16) = 31.53	P=0.5076	P=0.6784	P<0.0001
CDK5 cor	1.5	0.1586	0.7703	0.1755	1.435	0.125	0.8636	0.08677	0.0314	0.0009866	2.117	1.582	F (1, 16) = 0.3175	F (1, 16) = 0.009976	F (1, 16) = 21.41	P=0.5809	P=0.9217	P=0.0003
p-GSK3β/ GSK3β hip	1.392	0.1296	1.179	0.1342	1.228	0.1485	0.9448	0.06165	0.006135	0.1988	0.3078	1.213	F (1, 16) = 0.08092	F (1, 16) = 2.622	F (1, 16) = 4.059	P=0.7797	P=0.1250	P=0.0611
p-GSK3β/ GSK3β cor	1.219	0.1295	0.996	0.05909	1.14	0.2043	1.076	0.05951	0.03162	2.326e-006	0.1028	1.311	F (1, 16) = 0.3859	F (1, 16) = 2.839e-005	F (1, 16) = 1.255	P=0.5432	P=0.9958	P=0.2791
LC3 II hip	1.258	0.1491	0.8419	0.06745	1.324	0.1374	1.052	0.05322	0.02606	0.0949	0.5915	0.9697	F (1, 16) = 0.43	F (1, 16) = 1.566	F (1, 16) = 9.759	P=0.5213	P=0.2288	P=0.0065
LC3 II cor	1.177	0.03666	0.868	0.1232	1.206	0.1125	1.029	0.01838	0.02174	0.04543	0.2954	0.5905	F (1, 16) = 0.589	F (1, 16) = 1.231	F (1, 16) = 8.004	P=0.4540	P=0.2836	P=0.0121
p62 hip	1.336	0.09543	1.033	0.05545	1.355	0.08631	0.9903	0.04032	0.004647	0.0007535	0.5573	0.4251	F (1, 16) = 0.1749	F (1, 16) = 0.02836	F (1, 16) = 20.97	P=0.6813	P=0.8684	P=0.0003
p62 cor	1.029	0.03494	1.003	0.01168	1.11	0.04057	0.966	0.04739	0.01748	0.002418	0.03597	0.105	F (1, 16) = 2.665	F (1, 16) = 0.3685	F (1, 16) = 5.482	P=0.1221	P=0.5523	P=0.0325
p-mTOR/mTOR hip	0.3667	0.06207	0.7991	0.06862	0.3561	0.08319	0.881	0.0624	0.0107	0.006331	1.145	0.3875	F (1, 16) = 0.4417	F (1, 16) = 0.2614	F (1, 16) = 47.29	P=0.5158	P=0.6161	P<0.0001
p-mTOR/mTOR cor	0.1798	0.01824	0.7379	0.1174	0.5599	0.07564	1.107	0.1657	0.0001506	0.7016	1.527	0.946	F (1, 16) = 0.002547	F (1, 16) = 11.87	F (1, 16) = 25.83	P=0.9604	P=0.0033	P=0.0001
p-P70S6K/P70S6K hip	1.026	0.0333	1.744	0.2361	0.8132	0.1213	1.074	0.1027	0.2621	0.9742	1.2	1.642	F (1, 16) = 2.554	F (1, 16) = 9.492	F (1, 16) = 11.69	P=0.1296	P=0.0072	P=0.0035

p-P70S6K/P70S6K cor	0.7526	0.05115	1.107	0.1835	0.7335	0.1297	1.096	0.08829	6.692e-005	0.001198	0.6425	1.218	F (1, 16) = 0.0008791	F (1, 16) = 0.01573	F (1, 16) = 8.44	P=0.9767	P=0.9017	P=0.0103
Cyt C hip	1.344	0.2187	0.992	0.155	2.758	0.4361	1.033	0.1379	2.355	2.645	5.391	5.621	F (1, 16) = 6.704	F (1, 16) = 7.528	F (1, 16) = 15.35	P=0.0198	P=0.0144	P=0.0012
Cyt C cor	2.107	0.1925	1.121	0.1407	1.885	0.163	0.9422	0.1198	0.002264	0.2007	4.649	1.955	F (1, 16) = 0.01852	F (1, 16) = 1.643	F (1, 16) = 38.05	P=0.8934	P=0.2182	P<0.0001
COX IV hip	2.301	0.2791	1.755	0.212	2.95	0.3765	1.178	0.1284	1.88	0.006521	6.718	5.622	F (1, 16) = 5.351	F (1, 16) = 0.01856	F (1, 16) = 19.12	P=0.0343	P=0.8933	P=0.0005
COX IV cor	1.829	0.2629	1.159	0.07596	1.887	0.2941	1.021	0.1599	0.04791	0.007883	2.949	3.739	F (1, 16) = 0.205	F (1, 16) = 0.03373	F (1, 16) = 12.62	P=0.6568	P=0.8566	P=0.0027

Data were shown as the relative fold of N-Wt.

Supplementary Table 3. Summarized statistical analysis of immunostaining

	H-Tg		N-Tg		H-Wt		N-Wt		ANOVA SS				ANOVA F (DFn, DFd)			ANOVA P value		
	Mean	SEM	Mean	SEM	Mean	SEM	Mean	SEM	Interaction	Tg vs. Wt	H vs. N	Residual	Interaction	Tg vs. Wt	H vs. N	Interaction	Tg vs. Wt	H vs. N
p-tau T231 staining hip	782.3	82.23	71.67	32.3	640	126.7	57	32.75	12224	18487	1255180	149587	F (1, 8) = 0.6537	F (1, 8) = 0.9887	F (1, 8) = 67.13	P=0.4422	P=0.3492	P<0.0001
p-tau T231 staining cor	2367	237.9	935.3	167.6	1039	100.3	59.33	34.6	153680	3645416	4360896	575619	F (1, 8) = 2.136	F (1, 8) = 50.66	F (1, 8) = 60.61	P=0.1820	P=0.0001	P<0.0001
LC3 staining hip	603.1	28.09	300.4	28.29	326.6	36.42	127.9	39.02	12711	169421	169421	23476	F (1, 8) = 4.331	F (1, 8) = 57.73	F (1, 8) = 57.73	P=0.0710	P<0.0001	P<0.0001
LC3 staining cor	2136	101	859.7	98.76	1022	138.1	406	53.14	327030	1843184	2687587	251092	F (1, 8) = 10.42	F (1, 8) = 58.73	F (1, 8) = 85.63	P=0.0121	P<0.0001	P<0.0001

Data were shown as the integrated density of positive staining.

Supplementary Table 4. Summarized statistical analysis of Gene expression assessment

	H-Tg		N-Tg		H-Wt		N-Wt		ANOVA SS				ANOVA F (DFn, DFd)			ANOVA P value		
	Mean	SEM	Mean	SEM	Mean	SEM	Mean	SEM	Interaction	Tg vs. Wt	H vs. N	Residual	Interaction	Tg vs. Wt	H vs. N	Interaction	Tg vs. Wt	H vs. N
CD86 hip	1.784	0.1901	0.9782	0.09212	1.409	0.175	0.7951	0.09205	0.03686	0.3118	2.017	1.005	F (1, 12) = 0.4402	F (1, 12) = 3.723	F (1, 12) = 24.09	P=0.5196	P=0.0776	P=0.0004
CD86 cor	1.569	0.136	0.8928	0.1746	1.261	0.1205	0.8563	0.1138	0.07362	0.1186	1.168	0.9173	F (1, 12) = 0.9631	F (1, 12) = 1.551	F (1, 12) = 15.28	P=0.3458	P=0.2368	P=0.0021
CD206 hip	0.5676	0.124	1.424	0.2292	0.4138	0.1013	0.9169	0.1858	0.1249	0.437	1.849	1.352	F (1, 12) = 1.109	F (1, 12) = 3.878	F (1, 12) = 16.41	P=0.3131	P=0.0725	P=0.0016
CD206 cor	0.585	0.1507	1.617	0.311	0.4178	0.09286	1.147	0.1254	0.09182	0.4063	3.103	1.725	F (1, 12) = 0.6387	F (1, 12) = 2.826	F (1, 12) = 21.58	P=0.4397	P=0.1186	P=0.0006
IL-6 hip	5.203	0.6378	1.446	0.6811	3.149	0.4153	0.8631	0.1058	2.166	6.952	36.52	12.65	F (1, 12) = 2.055	F (1, 12) = 6.594	F (1, 12) = 34.64	P=0.1773	P=0.0246	P<0.0001
IL-6 cor	6.729	0.6336	2.308	0.6734	6.365	0.9277	1.459	0.2392	0.2351	1.472	87	21.27	F (1, 12) = 0.1326	F (1, 12) = 0.8305	F (1, 12) = 49.08	P=0.7221	P=0.3801	P<0.0001
TNF-α hip	2.579	0.3651	0.9628	0.1449	2.333	0.2472	1.13	0.07199	0.1702	0.006137	7.946	2.647	F (1, 12) = 0.7715	F (1, 12) = 0.02782	F (1, 12) = 36.02	P=0.3970	P=0.8703	P<0.0001
TNF-α cor	1.852	0.3408	0.3087	0.0931	2.662	0.4678	0.8595	0.1071	0.06706	1.851	11.2	4.261	F (1, 12) = 0.1888	F (1, 12) = 5.213	F (1, 12) = 31.54	P=0.6716	P=0.0414	P=0.0001
IL-4 hip	0.3993	0.1387	1.627	0.292	0.1107	0.03196	1.004	0.1187	0.1113	0.8296	4.498	1.435	F (1, 12) = 0.931	F (1, 12) = 6.937	F (1, 12) = 37.61	P=0.3537	P=0.0218	P<0.0001
IL-4 cor	0.4494	0.1642	2.246	0.3784	0.2013	0.08351	1.148	0.2483	0.7217	1.811	7.523	2.866	F (1, 12) = 3.022	F (1, 12) = 7.583	F (1, 12) = 31.5	P=0.1077	P=0.0175	P=0.0001
IL-10 hip	0.246	0.1375	1.109	0.1678	0.3018	0.08104	1.063	0.1531	0.01037	9.914e-005	2.639	0.9249	F (1, 12) = 0.1345	F (1, 12) = 0.001286	F (1, 12) = 34.24	P=0.7202	P=0.9720	P<0.0001
IL-10 cor	0.04252	0.008212	0.9343	0.2125	0.2263	0.06302	1.148	0.2068	0.0009259	0.1584	3.29	1.104	F (1, 12) = 0.01006	F (1, 12) = 1.722	F (1, 12) = 35.77	P=0.9217	P=0.2140	P<0.0001
CCL2 hip	11.07	1.967	0.8595	0.08972	5.334	0.9544	0.6601	0.1284	30.65	35.23	221.5	57.68	F (1, 12) = 6.377	F (1, 12) = 7.329	F (1, 12) = 46.09	P=0.0266	P=0.0191	P<0.0001
CCL2 cor	7.725	0.3552	0.78	0.06251	13.39	3.026	0.6239	0.1952	33.86	30.33	388.4	111.9	F (1, 12) = 3.632	F (1, 12) = 3.253	F (1, 12) = 41.66	P=0.0809	P=0.0965	P<0.0001
CCL3 hip	12.51	2.009	1.803	0.1477	12.38	2.884	0.682	0.1666	0.9783	1.572	501.9	148.9	F (1, 12) = 0.07886	F (1, 12) = 0.1267	F (1, 12) = 40.46	P=0.7836	P=0.7281	P<0.0001
CCL3 cor	10.32	1.524	2.801	0.6184	9.414	2.887	0.988	0.5917	0.8319	7.371	254.1	136.7	F (1, 12) = 0.07302	F (1, 12) = 0.647	F (1, 12) = 22.3	P=0.7916	P=0.4368	P=0.0005

Data were shown as the relative fold of N-Wt.
