# Supplementary material for: Impacts of Acute Hypoxia on Alzheimer's Disease-Like Pathologies in APPswe/PS1dE9 Mice and Their Wild Type Littermates
Source: Front Neurosci. 2018 May 9;12:314. doi: 10.3389/fnins.2018.00314 (PMC5954115; doi:10.3389/fnins.2018.00314)
Supplement: Supplementary file 4 [file Data_Sheet_1.PDF]

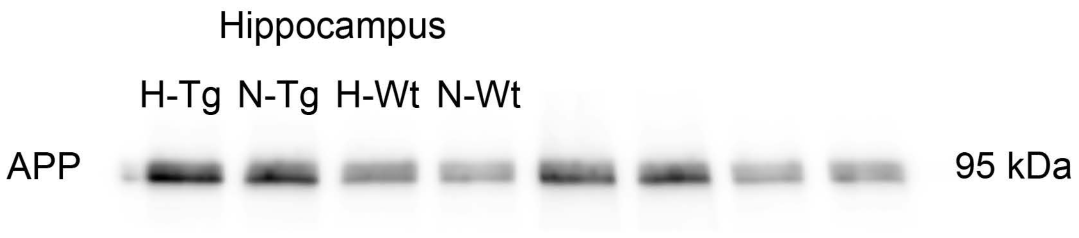
Hippocampus
H-Tg
N-Tg
H-Wt
N-Wt
APP
95 kDa

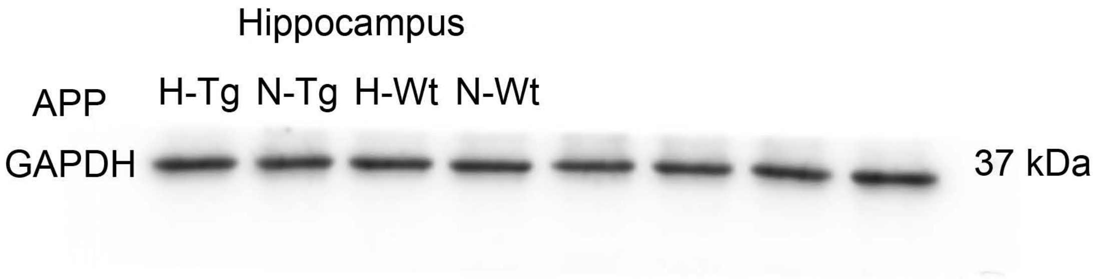
Hippocampus
APP
H-Tg
N-Tg
H-Wt
N-Wt
GAPDH
37 kDa

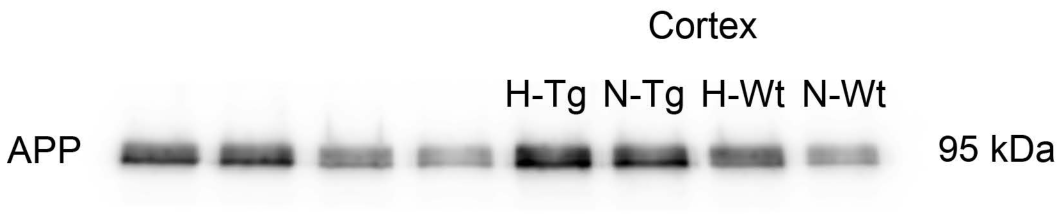
Cortex
H-Tg
N-Tg
H-Wt
N-Wt
APP
95 kDa

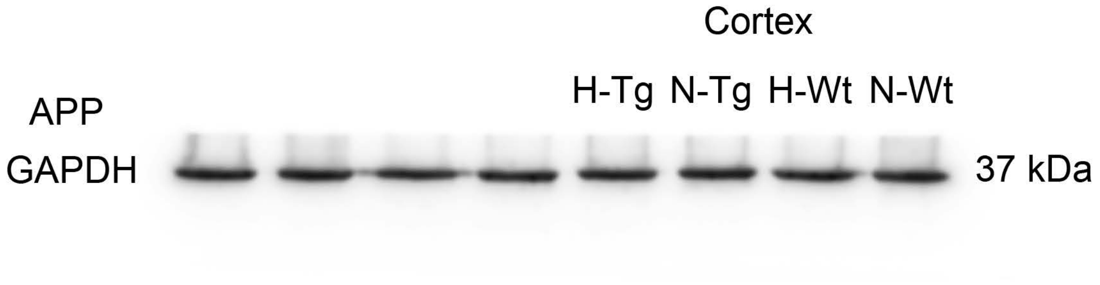

Cortex
H-Tg
N-Tg
H-Wt
N-Wt
APP
GAPDH
37 kDa

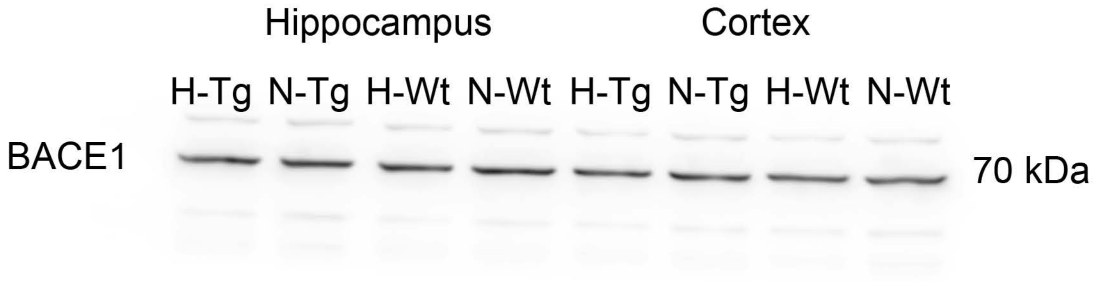
Hippocampus
Cortex
H-Tg
N-Tg
H-Wt
N-Wt
H-Tg
N-Tg
H-Wt
N-Wt
BACE1
70 kDa

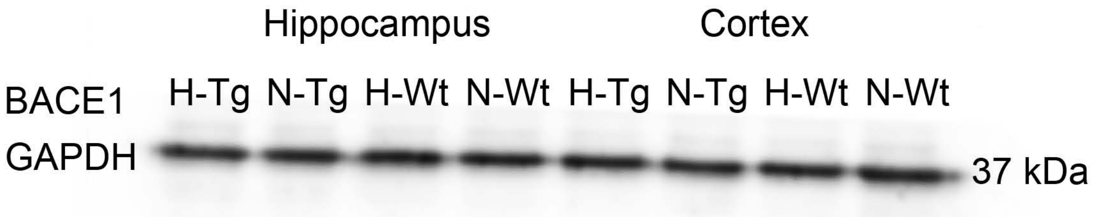
Hippocampus
Cortex
BACE1
H-Tg N-Tg H-Wt N-Wt H-Tg N-Tg H-Wt N-Wt
GAPDH
37 kDa

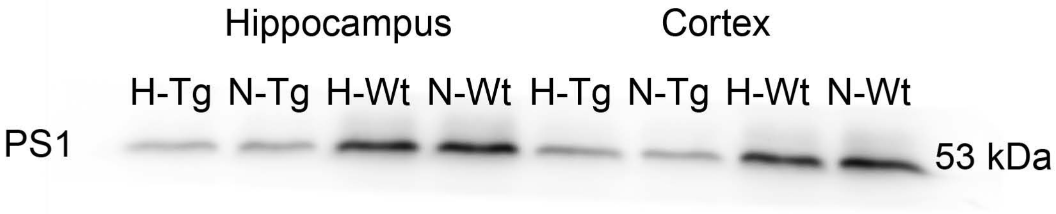
Hippocampus
Cortex
H-Tg
N-Tg
H-Wt
N-Wt
H-Tg
N-Tg
H-Wt
N-Wt
PS1
53 kDa

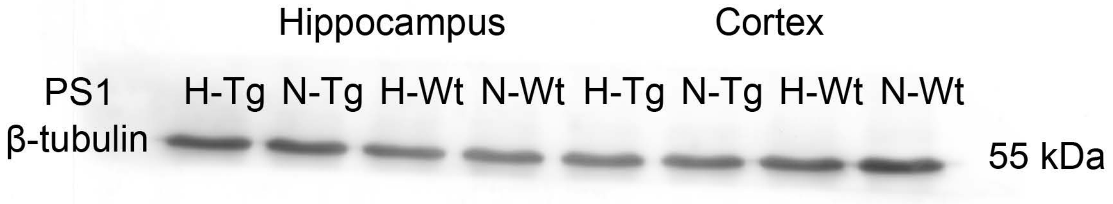
Hippocampus
Cortex
PS1
H-Tg N-Tg H-Wt N-Wt H-Tg N-Tg H-Wt N-Wt
β-tubulin
55 kDa

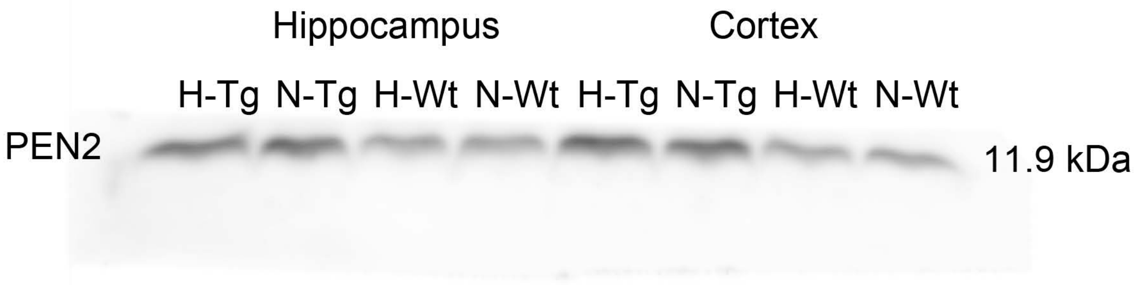
Hippocampus
Cortex
H-Tg N-Tg H-Wt N-Wt H-Tg N-Tg H-Wt N-Wt
PEN2
11.9 kDa

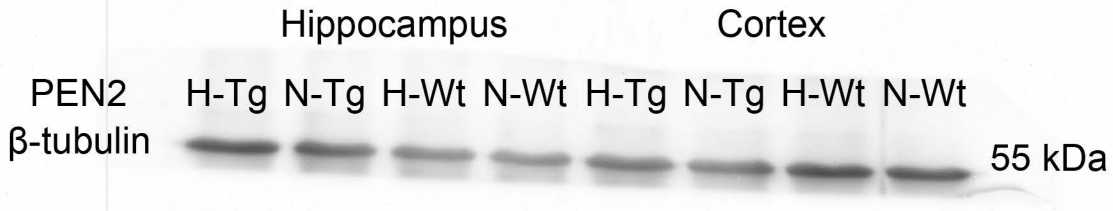
Hippocampus
Cortex
PEN2
β-tubulin
H-Tg
N-Tg
H-Wt
N-Wt
H-Tg
N-Tg
H-Wt
N-Wt
55 kDa

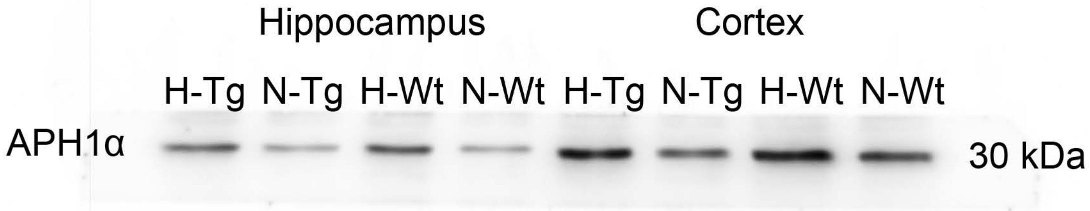
Hippocampus
Cortex
H-Tg
N-Tg
H-Wt
N-Wt
H-Tg
N-Tg
H-Wt
N-Wt
APH1α
30 kDa

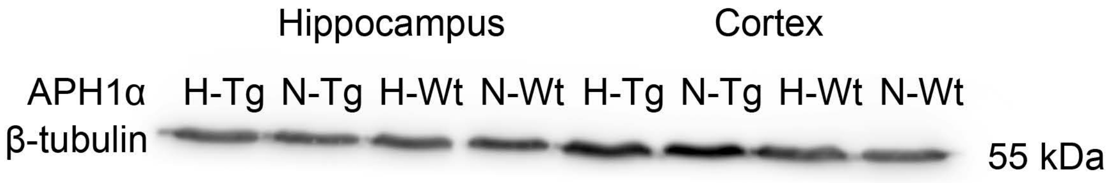
Hippocampus
Cortex
APH1α
H-Tg
N-Tg
H-Wt
N-Wt
H-Tg
N-Tg
H-Wt
N-Wt
β-tubulin
55 kDa

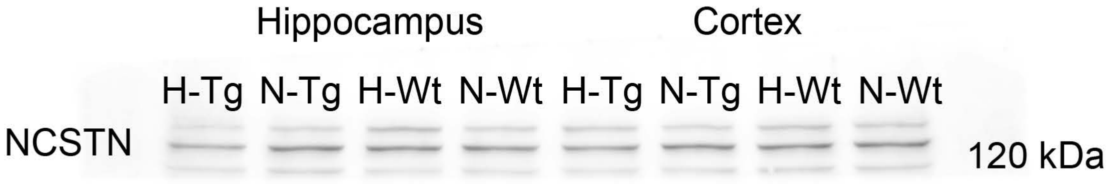
Hippocampus
Cortex
H-Tg
N-Tg
H-Wt
N-Wt
H-Tg
N-Tg
H-Wt
N-Wt
NCSTN
120 kDa

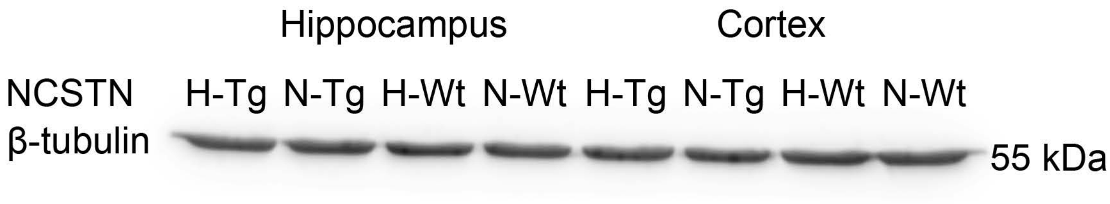
Hippocampus
Cortex
NCSTN
H-Tg
N-Tg
H-Wt
N-Wt
H-Tg
N-Tg
H-Wt
N-Wt
β-tubulin
55 kDa

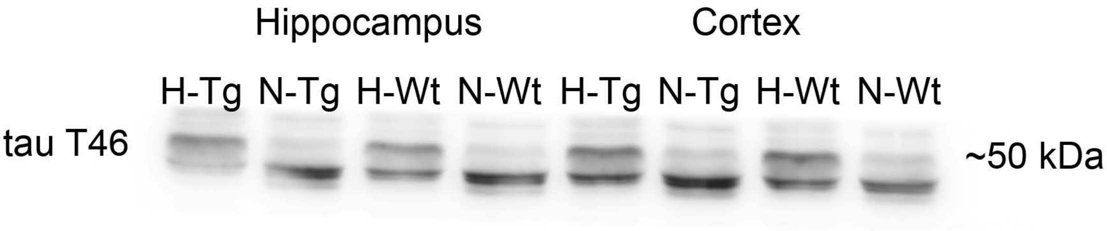
Hippocampus
Cortex
H-Tg
N-Tg
H-Wt
N-Wt
H-Tg
N-Tg
H-Wt
N-Wt
tau T46
~50 kDa

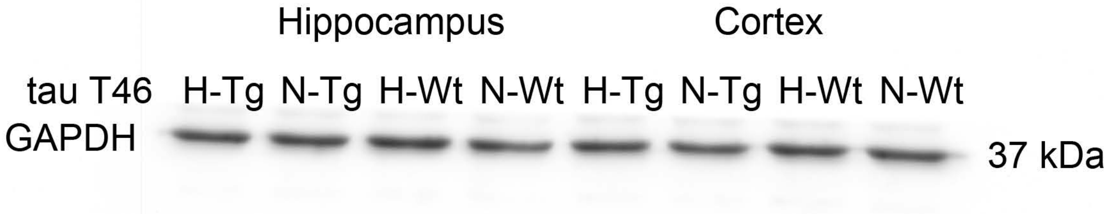
Hippocampus
Cortex
tau T46
H-Tg
N-Tg
H-Wt
N-Wt
H-Tg
N-Tg
H-Wt
N-Wt
GAPDH
37 kDa

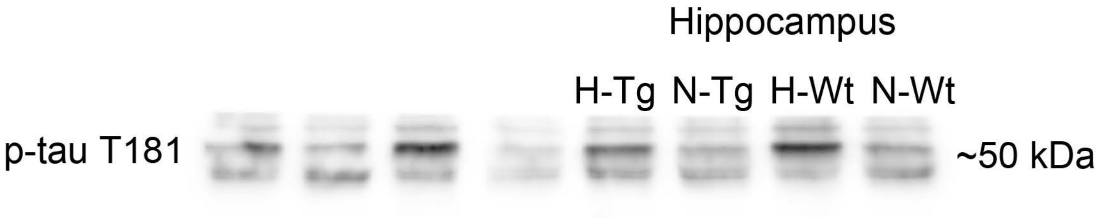
Hippocampus
H-Tg N-Tg H-Wt N-Wt
p-tau T181
~50 kDa

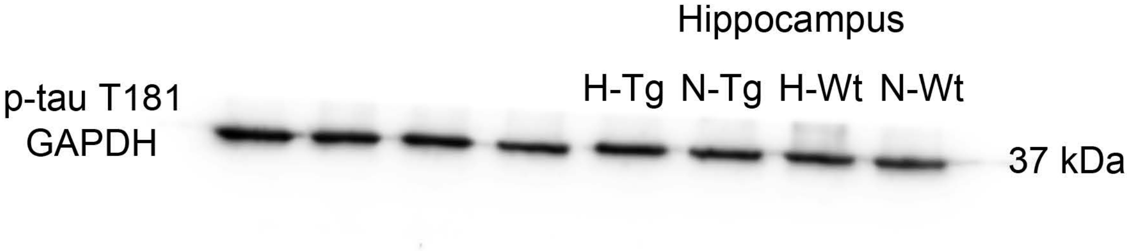
Hippocampus
H-Tg
N-Tg
H-Wt
N-Wt
p-tau T181
GAPDH
37 kDa

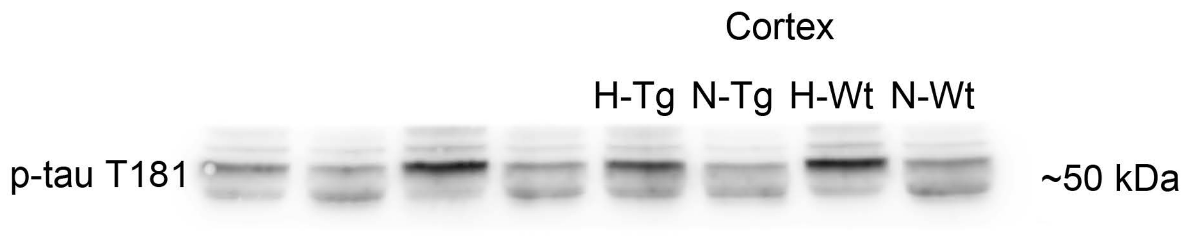
Cortex
H-Tg
N-Tg
H-Wt
N-Wt
p-tau T181
~50 kDa

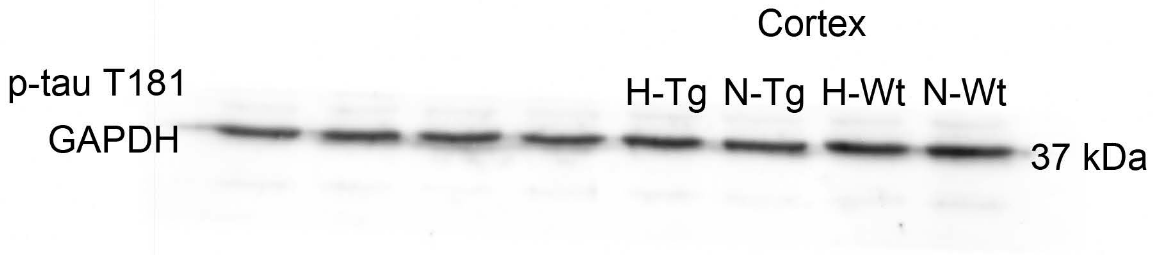
Cortex
p-tau T181
H-Tg N-Tg H-Wt N-Wt
GAPDH
37 kDa

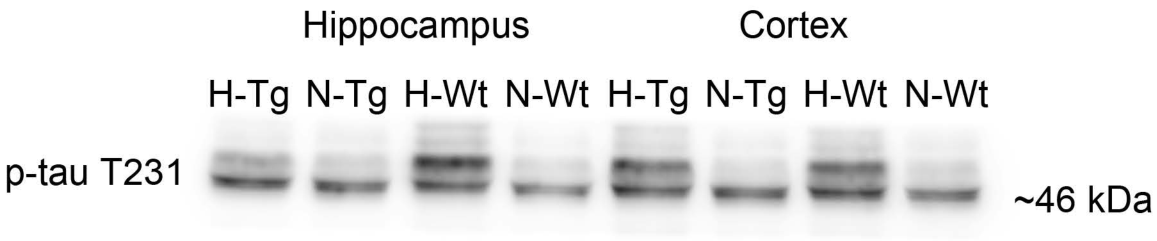
Hippocampus
Cortex
H-Tg
N-Tg
H-Wt
N-Wt
H-Tg
N-Tg
H-Wt
N-Wt
p-tau T231
~46 kDa

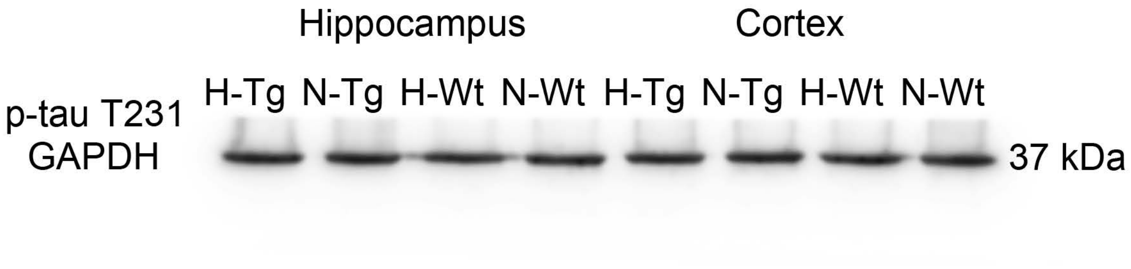
Hippocampus
Cortex
H-Tg N-Tg H-Wt N-Wt H-Tg N-Tg H-Wt N-Wt
p-tau T231
GAPDH
37 kDa

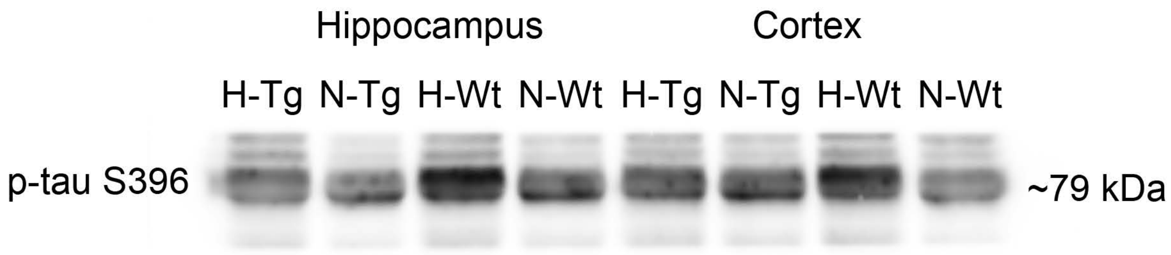
Hippocampus
Cortex
H-Tg
N-Tg
H-Wt
N-Wt
H-Tg
N-Tg
H-Wt
N-Wt
p-tau S396
~79 kDa

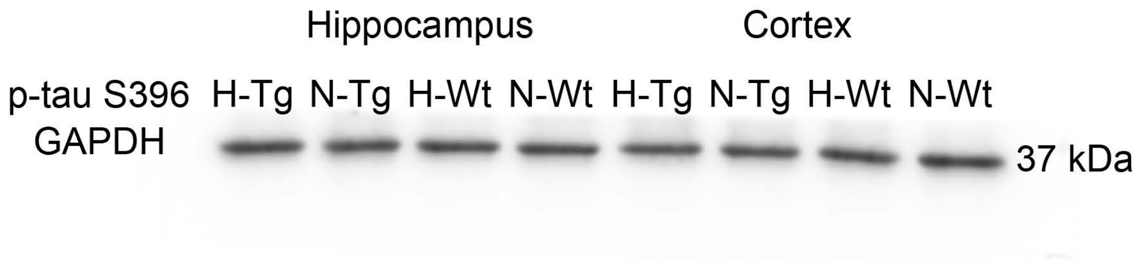

Hippocampus
Cortex
p-tau S396
H-Tg N-Tg H-Wt N-Wt H-Tg N-Tg H-Wt N-Wt
GAPDH
37 kDa

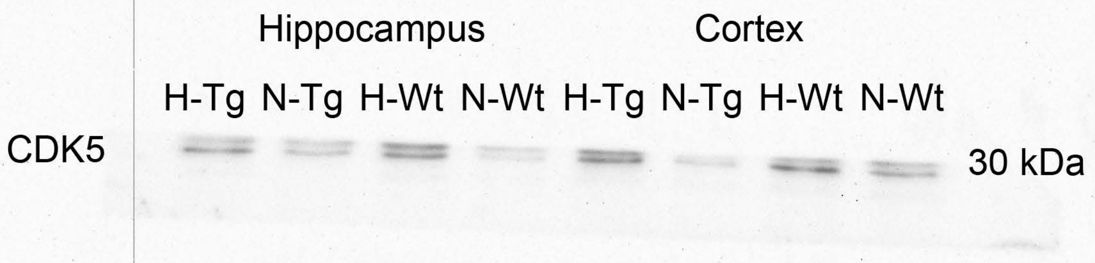
Hippocampus
Cortex
H-Tg
N-Tg
H-Wt
N-Wt
H-Tg
N-Tg
H-Wt
N-Wt
CDK5
30 kDa

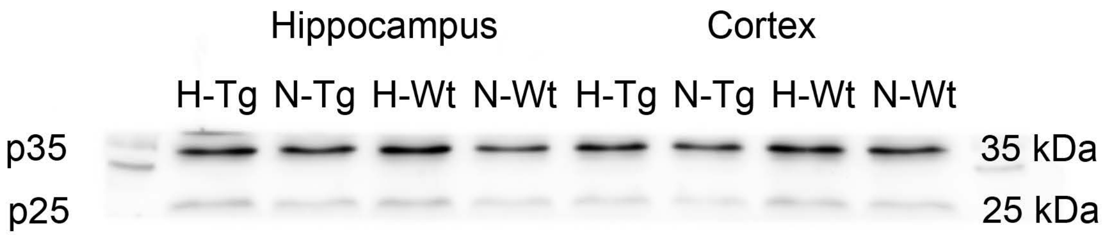
Hippocampus
Cortex
H-Tg
N-Tg
H-Wt
N-Wt
H-Tg
N-Tg
H-Wt
N-Wt
p35
p25
35 kDa
25 kDa

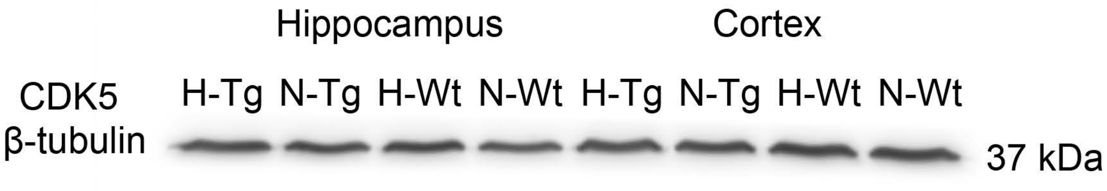
Hippocampus
Cortex
CDK5
β-tubulin
H-Tg N-Tg H-Wt N-Wt H-Tg N-Tg H-Wt N-Wt
37 kDa

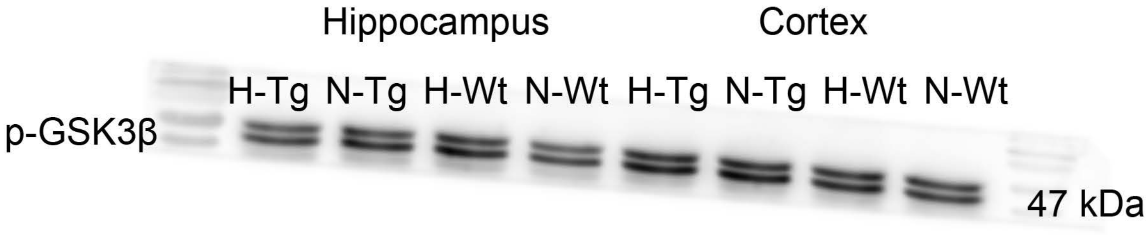
Hippocampus
Cortex
H-Tg N-Tg H-Wt N-Wt H-Tg N-Tg H-Wt N-Wt
p-GSK3β
47 kDa

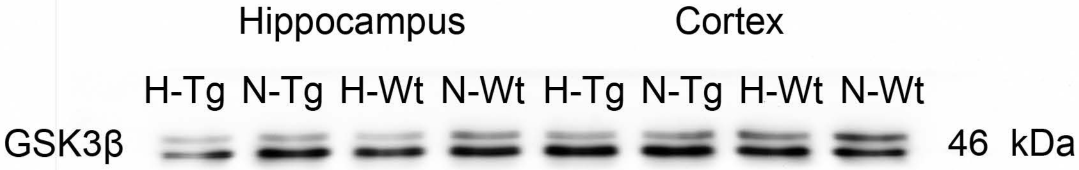
Hippocampus
Cortex
H-Tg
N-Tg
H-Wt
N-Wt
H-Tg
N-Tg
H-Wt
N-Wt
GSK3β
46 kDa

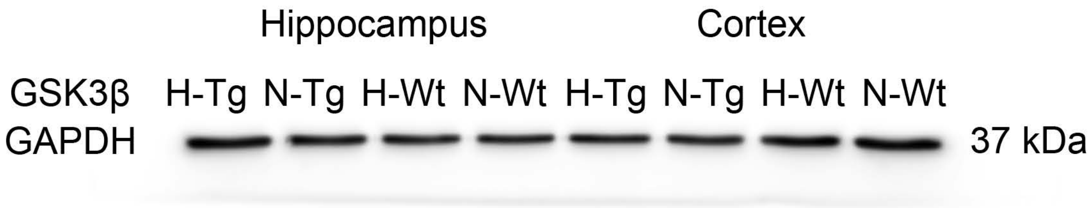
Hippocampus
Cortex
GSK3β
H-Tg
N-Tg
H-Wt
N-Wt
H-Tg
N-Tg
H-Wt
N-Wt
GAPDH
37 kDa

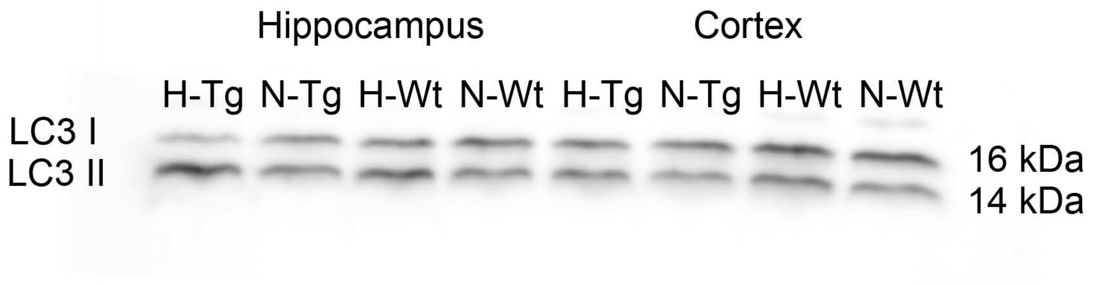
Hippocampus
Cortex
H-Tg
N-Tg
H-Wt
N-Wt
H-Tg
N-Tg
H-Wt
N-Wt
LC3 I
LC3 II
16 kDa
14 kDa

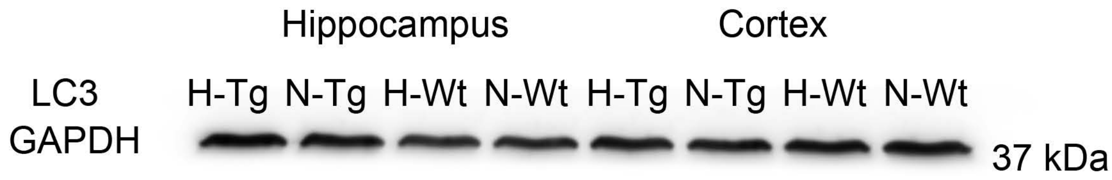
Hippocampus
Cortex
LC3
H-Tg N-Tg H-Wt N-Wt H-Tg N-Tg H-Wt N-Wt
GAPDH
37 kDa

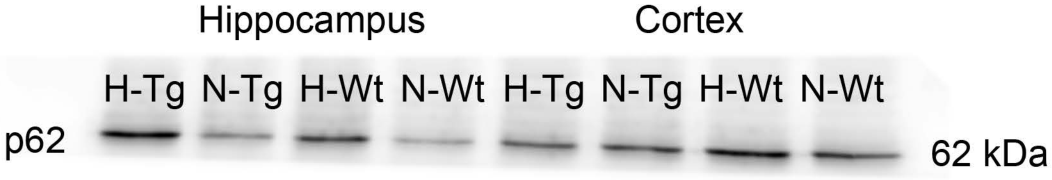

Hippocampus
Cortex
H-Tg
N-Tg
H-Wt
N-Wt
H-Tg
N-Tg
H-Wt
N-Wt
p62
62 kDa

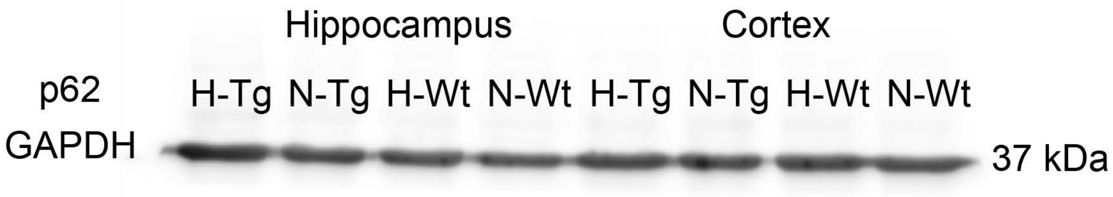

Hippocampus
Cortex
p62
H-Tg N-Tg H-Wt N-Wt H-Tg N-Tg H-Wt N-Wt
GAPDH
37 kDa

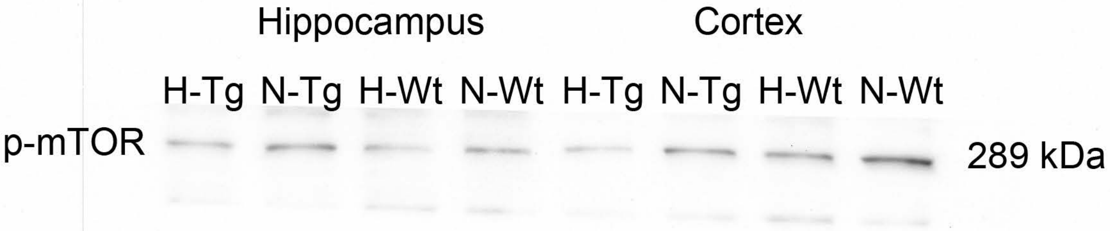
Hippocampus
Cortex
H-Tg
N-Tg
H-Wt
N-Wt
H-Tg
N-Tg
H-Wt
N-Wt
p-mTOR
289 kDa

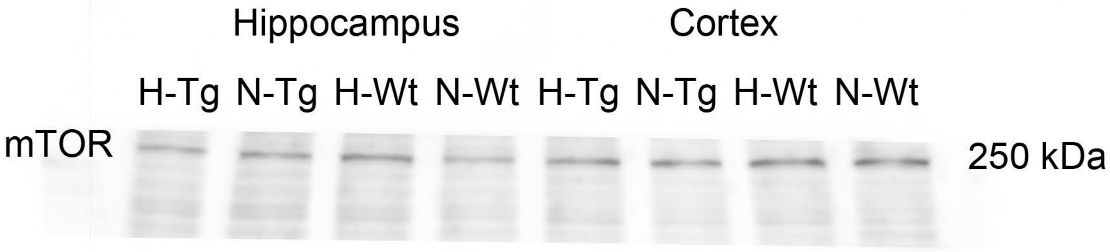
Hippocampus
Cortex
H-Tg
N-Tg
H-Wt
N-Wt
H-Tg
N-Tg
H-Wt
N-Wt
mTOR
250 kDa

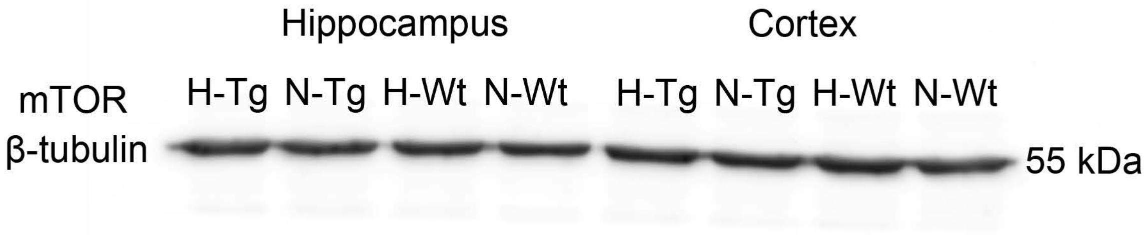
Hippocampus
Cortex
mTOR
H-Tg
N-Tg
H-Wt
N-Wt
H-Tg
N-Tg
H-Wt
N-Wt
β-tubulin
55 kDa

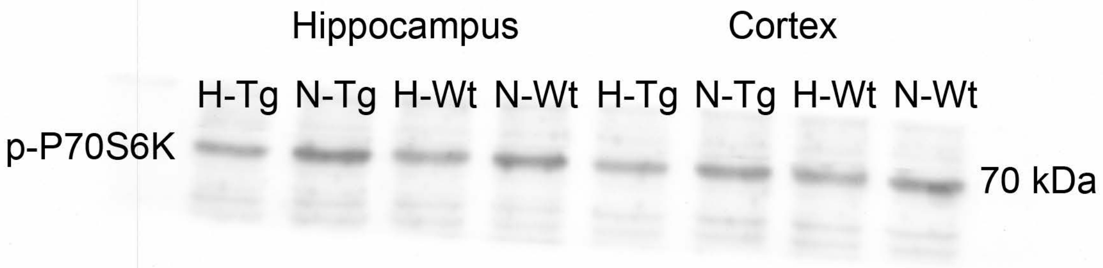
Hippocampus
Cortex
H-Tg N-Tg H-Wt N-Wt H-Tg N-Tg H-Wt N-Wt
p-P70S6K
70 kDa

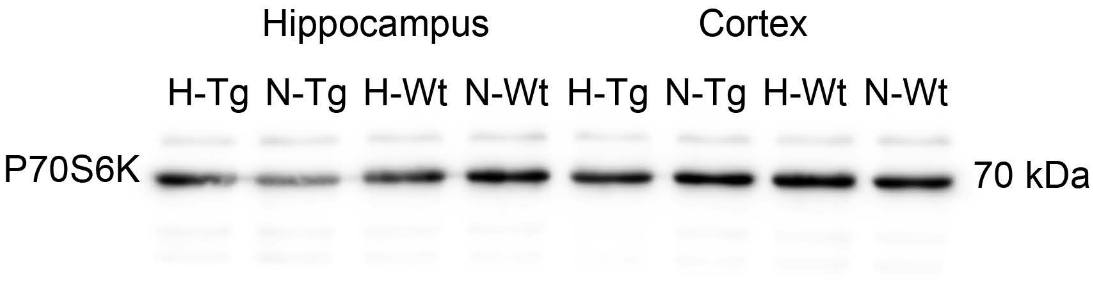
Hippocampus
Cortex
H-Tg
N-Tg
H-Wt
N-Wt
H-Tg
N-Tg
H-Wt
N-Wt
P70S6K
70 kDa

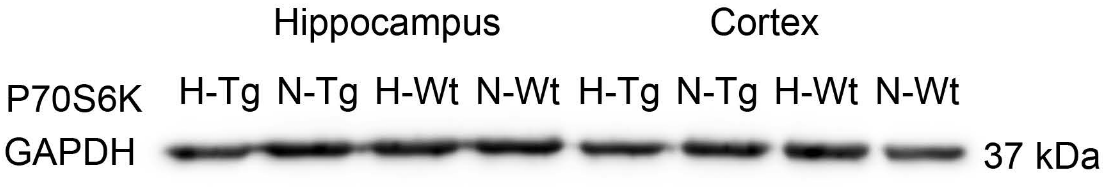
Hippocampus
Cortex
P70S6K
H-Tg N-Tg H-Wt N-Wt H-Tg N-Tg H-Wt N-Wt
GAPDH
37 kDa

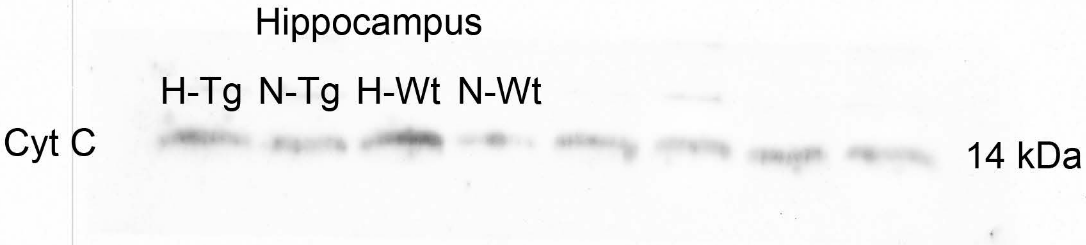
Hippocampus
H-Tg N-Tg H-Wt N-Wt
Cyt C
14 kDa

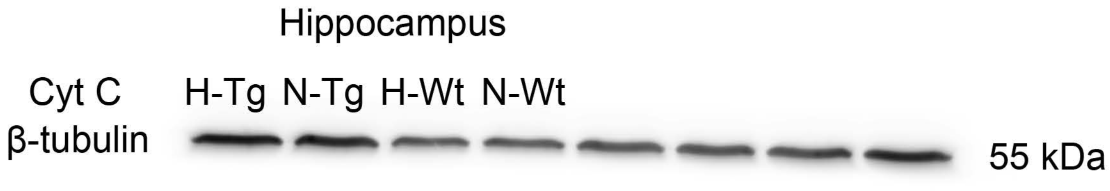
Hippocampus
Cyt C
β-tubulin
H-Tg
N-Tg
H-Wt
N-Wt
55 kDa

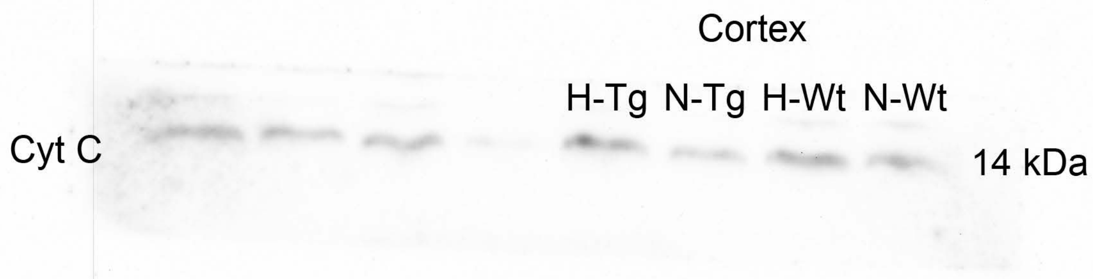
Cortex
H-Tg N-Tg H-Wt N-Wt
Cyt C
14 kDa

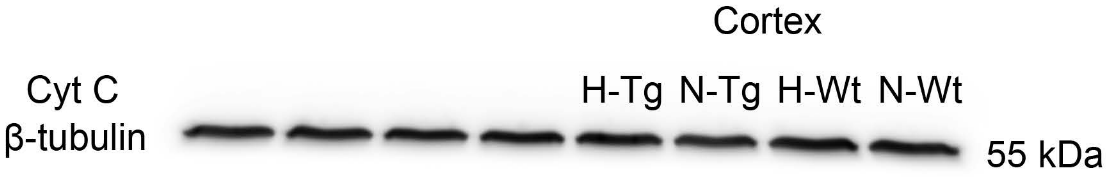
Cortex
Cyt C
β-tubulin
H-Tg
N-Tg
H-Wt
N-Wt
55 kDa

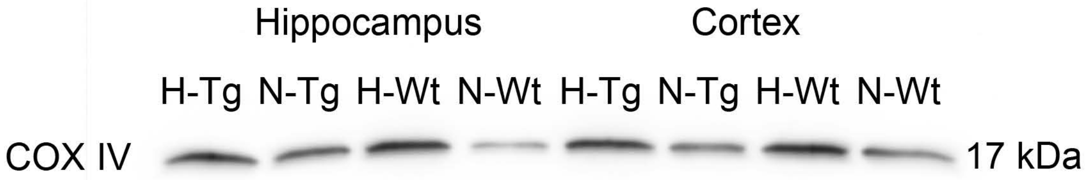
Hippocampus
Cortex
H-Tg
N-Tg
H-Wt
N-Wt
H-Tg
N-Tg
H-Wt
N-Wt
COX IV
17 kDa

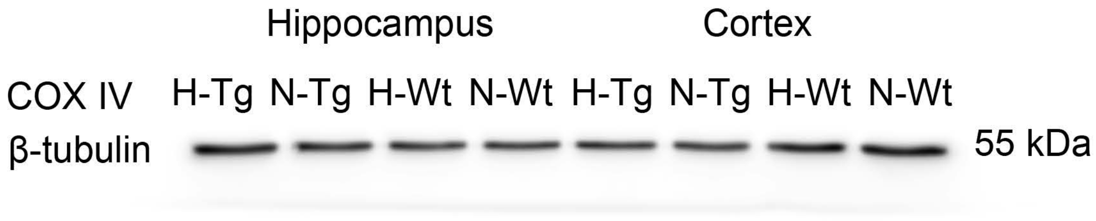
Hippocampus
Cortex
COX IV
H-Tg N-Tg H-Wt N-Wt H-Tg N-Tg H-Wt N-Wt
β-tubulin
55 kDa
